# Dietary and environmental factors have opposite AhR-dependent effects on *C. elegans* healthspan

**DOI:** 10.18632/aging.202316

**Published:** 2020-12-13

**Authors:** Vanessa Brinkmann, Alfonso Schiavi, Anjumara Shaik, Daniel Rüdiger Puchta, Natascia Ventura

**Affiliations:** 1 Leibniz Institute for Environmental Medicine, Auf’m Hennekamp 50, 40225 Düsseldorf, Germany; 2 Institute of Clinical Chemistry and Laboratory Diagnostic, Heinrich Heine University Düsseldorf, Moorenstr 5, 40225 Düsseldorf, Germany

**Keywords:** aryl hydrocarbon receptor, *C. elegans*, microbiota, environment, aging

## Abstract

Genetic, dietary, and environmental factors concurrently shape the aging process. The aryl hydrocarbon receptor (AhR) was discovered as a dioxin-binding transcription factor involved in the metabolism of different environmental toxicants in vertebrates. Since then, the variety of pathophysiological processes regulated by the AhR has grown, ranging from immune response, metabolic pathways, and aging. Many modulators of AhR activity may impact on aging and age-associated pathologies, but, whether their effects are AhR-dependent has never been explored. Here, using *Caenorhabditis elegans*, as an elective model organism for aging studies, we show for the first time that lack of *Ce*AHR-1 can have opposite effects on health and lifespan in a context-dependent manner. Using known mammalian AhR modulators we found that, *ahr-1* protects against environmental insults (benzo(a)pyrene and UVB light) and identified a new role for AhR-bacterial diet interaction in animal lifespan, stress resistance, and age-associated pathologies. We narrowed down the dietary factor to a bacterially extruded metabolite likely involved in tryptophan metabolism. This is the first study clearly establishing *C. elegans* as a good model organism to investigate evolutionarily conserved functions of AhR-modulators and -regulated processes, indicating it can be exploited to contribute to the discovery of novel information about AhR in mammals.

## INTRODUCTION

Aging affects every human and is accompanied by increased morbidity (e.g., diabetes, cardiovascular diseases, neurodegenerative diseases, and cancer) and risk of death [1, 2]. In the past decades, also thanks to the growing number of researchers exploiting simple but powerful model organisms such as the nematode *Caenorhabditis elegans* (*C. elegans*), numerous hallmarks of aging as well as genetic and environmental factors regulating it have been identified [2–4]. The aryl hydrocarbon receptor (AhR) is a highly conserved transcription factor of the basic helix–loop–helix/PER–ARNT–SIM (bHLH/PAS) family originally discovered as a dioxin-binding protein in vertebrates [5] and involved in the metabolism of different environmental toxicants and xenobiotics. Since its discovery, the variety of pathophysiological processes regulated by the AhR has rapidly grown and range from cell death, to immune response and neuronal development [6]. Contradictory studies also indicated a role for AhR in the aging process (reviewed in [7]) and more recently, pro-aging functions of AhR [8, 9] have been described in an evolutionarily conserved manner from *C. elegans* to mammals. In mammals, in basal conditions, AhR is bound to heat shock protein 90 (HSP90), AhR interacting protein (AIP), and p23, which retain it in the cytoplasm, in a ligand-affine state. Ligand binding of an AhR agonist leads to the dissociation of the AhR binding complex and AhR nuclear translocation [10, 11]. In the nucleus, AhR dimerizes with the AhR nuclear translocator (Arnt), and the AhR-Arnt heterodimer then binds to the xenobiotic responsive elements (XREs) on AhR target genes [11]. Similar to its mammalian counterpart, the *C. elegans* AhR homolog, AHR-1 (or CeAhR), forms a heterodimer with the *C. elegans* Arnt homolog AHA-1 and binds to XREs [12]. AHR-1 is expressed in several types of neurons and plays a role in neurodevelopment and long-chain unsaturated fatty acid synthesis [13–17]. However, unlike mammalian AhR it does not bind to typical activators such as 2,3,7,8-tetrachlorodibenzodioxin (TCDD) or β-naphthoflavone [12] suggesting that the xenobiotic-metabolizing activity of the receptor may not be an ancestral function or that its xenobiotic binding activity has been lost during nematode evolution. Although AhR was originally discovered as a dioxin-binding protein [5] in more recent years the number of compounds which modulate its activity and the consequent downstream responses (reviewed in [18]), has significantly expanded and can be broadly divided into four categories: xenobiotics (e.g., TCDD and benzo(a)pyrene (BaP)), dietary factors (e.g., kaempferol, curcumin), endogenous modulators (e.g., 6-formylindolo[3,2-b]carbazole (FICZ) and kynurenine) and ligands generated by the microbiota metabolism (e.g., indole-3-acetate and tryptamine) [5, 19–25]. Interestingly, many of these AhR modulators may impact on aging and age-associated pathologies [26–28], but whether these effects are AhR-dependent and whether AhR itself influences the progression of these diseases has been largely unexplored. Given the inability of CeAhR to bind classical ligands, *C. elegans* represents an ideal system to unravel novel physiological functions of AhR in the presence or absence of other possible modulators of its activity.

Here, we followed up on our previous finding indicating an anti-aging effect of *C. elegans* *ahr-1* depletion [8] and investigated the effect of possible AHR-1 modulators on aging and age-related features, namely stress response and neuromuscular pathologies. Our findings indicate that *C. elegans* can be exploited to study evolutionarily conserved functions of AhR modulators and their regulated processes and therefore to contribute to the discovery of novel information about AhR plasticity in mammals. Most importantly, we identify aging and associated pathologies as novel life traits regulated by the AhR in a microbiota-dependent manner bringing further complexity to the landscape of AhR-microbiota regulated processes.

## RESULTS

### *C. elegans* AHR-1 is differentially involved in stress response

**Figure 1:**
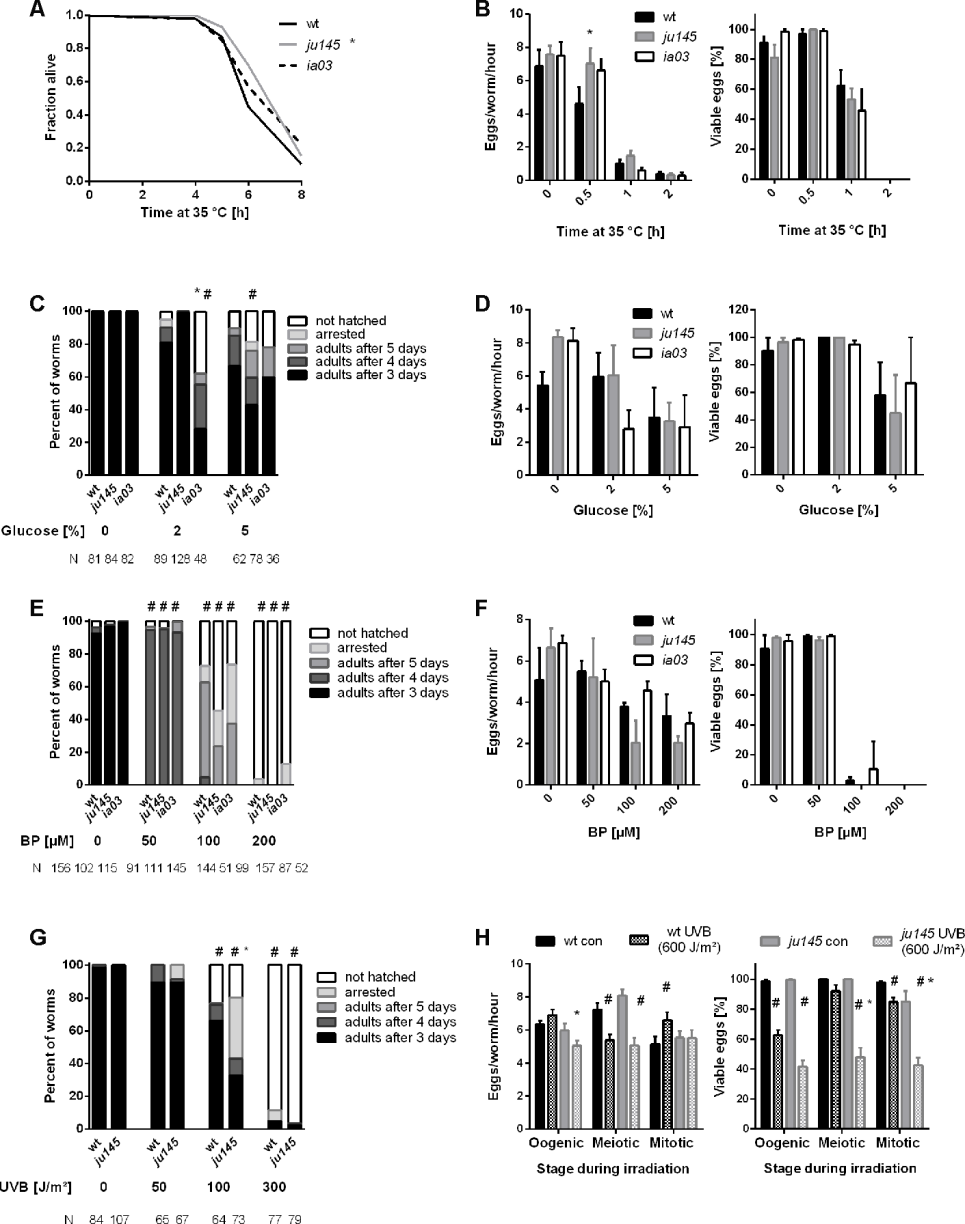
**Loss of *ahr-1* differentially regulates resistance to stressors.** (**A**) Survival in response to heat shock. Curves show the pooled data of 60 worms in 3 independent replicates. Statistical test: Log-Rank test, * significance *vs*. wild-type. (**B**) Fertility after heat stress. Shown are the number (left panel) and viability (right panel) of eggs laid from gravid adults treated with heat shock for the indicated time. Mean + SEM of pooled data from 18 worms/condition in 3 independent experiments are shown. (**C**, **D**) Development and fertility in response to the indicated concentration of glucose. Means (+SEM) of 3 independent replicates are shown. N = number of individuals in panel (**C**), 9 individuals were used in panel (**D**). (**E**, **F**) Development and fertility in response to the indicated concentration of iron chelator (BP). Means (+SEM) of 3 and 4 independent replicates are shown. N = number of individuals in panel (**E**), 9 individuals were used in panel (**F**). (**G**, **H**) Development and fertility in response to indicated doses of UVB. Fertility was assessed at a dose of 600 J/m². Means (+SEM) of 3 independent replicates are shown. N = number of individuals in panel (**G**), 9 individuals were used in panel (**H**)**.** (**B**–**H**) Statistical test: 2-way ANOVA with Tukey’s multiple comparisons test, * significance *vs.* wild-type, # significance *vs.* control (untreated), p-value < 0.05.

Loss of AHR-1 extends *C. elegans’* health- and lifespan [8]. Since lifespan extension often correlates with increased resistance to different types of stressors [29], we assessed AhR role in stress resistance by exposing wild-type and *ahr-1* mutants to either heat shock, metabolic stressors (i.e. high concentrations of glucose or the hypoxia mimetic iron chelator 2,2’Bipyridyl, BP) or UVB light. As expected, the development and fertility of wild-type animals were affected by all insults in a dose-dependent manner (Figure 1) [30, 31]. Consistent with our previous data [8] loss of *ahr-1* increased heat-stress resistance (Figure 1A, 1B), but it did not confer resistance to any of the newly tested insults (Figure 1C–1H). Actually, UVB-induced developmental delay and embryonic lethality were significantly more affected in the *ahr-1(ju145)* mutants than in wild-type worms (Figure 1G, 1H). To further characterize animals’ stress response, we quantified the expression of *C. elegans* transgenic reporters for different phase-I and phase-II genes typically regulated by AhR in mammals: *cyps* (*e.g.*, *CYP1A1* or *CYP1B1*), *ugts* (*e.g.,* *UGT1A1* or *UGT1A6*), and *gsts* (*e.g.,* *GSTA1* or *GSTA2*) [11, 32, 33]. Out of nine tested reporters, we found five genes (*cyp-35A2*, *cyp-35B1,* *gst-4, cyp-37A1,* and *ugt-29*) differentially expressed by at least ten percent upon *ahr-1* RNAi (Supplementary Figure 1; Supplementary Table 1). Unexpectedly, when crossed into the *ahr-1(ju145)* mutant background, we observed an opposite effect on *cyp-35B1* while the *gst-4* and the *ugt-29* expression remained increased and reduced respectively in the *ahr-1* mutant (Supplementary Figure 1C, 1D, 1E). We previously showed that the *ahr-1* mutant lives longer than wild-type [8] and in line with the differential effect between *ahr-1* RNAi and the mutant, we now found that *ahr-1* RNAi does not extend the lifespan of the wild-type animals (Table 1; Supplementary Figure 1F). These data suggest either different modes of action of *ahr-1* silencing and mutation or tissue-specific effects disclosed by the RNAi treatment. Of note, while in most cases tissue-specific *ahr-1* RNAi had no effects, health- and lifespan were significantly shortened when RNAi was enhanced in the nervous system (Table 1; Supplementary Figure 1G). Taken together results described so far reveal a complex AHR-1 role in *C. elegans’* lifespan and response to stress in a tissue- and insult-dependent manner.

**Table 1:** Survival analysis upon tissue-specific *ahr-1* RNAi.

**Condition**	**Genotype**	**Description**	**Lifespan and healthspan (days)**	**N**	**n**	**P-value**
N2 con			19.88 ± 0.29 16.00 ± 0.25	280	5	
N2 *ahr-1*	wild-type	wild-type	19.46 ± 0.29 15.33 ± 0.25	264	5	0.14 0.04
NR222 *con*	rde-1(ne219); kzIs9 [lin-26p::nls::GFP + lin-26p::rde-1) + rol-6(su1006)]	hypodermis-specific RNAi [88]	19.07 ± 0.34 15.12 ± 0.26	120	2	
NR222 *ahr-1*			18.78 ± 0.30 15.21 ± 0.29	120	2	0.23 0.65
NL2098 con	rrf-1(pk1417)	germline-specific RNAi [89, 90]	18.26 ± 0.45 14.16 ± 0.34	120	2	
NL2098 *ahr-1*			18.24 ± 0.43 13.98 ± 0.34	120	2	0.83 0.89
NL2550 con	ppw-1(pk2505)	RNAi only in somatic tissues [91]	19.51 ± 0.59 15.92 ± 0.53	120	2	
NL2550 *ahr-1*			18.78 ± 0.46 15.25 ± 0.40	120	2	0.11 0.19
WM118 con	rde-1(ne300); neIs9 [myo-3::HA::RDE-1+ pRF4(rol-6(su1006)]	muscle-specific RNAi [92]	15.49 ± 0.45 11.91 ± 0.29	120	2	0.92 0.27
WM118 *ahr-1*			15.40 ± 0.45 11.44 ± 0.29	120	2	
VP303 con	rde-1(ne219); kbIs7 [nhx-2p::rde-1 + rol-6(su1006)]	intestine-specific RNAi [93]	18.68 ± 0.41 16.52 ± 0.33	120	2	0.47 0.72
VP303 *ahr-1*			18.50 ± 0.37 16.78 ± 0.32	120	2	
TU3401 con	sid-1(pk3321); uls69[myo-2p::mCherry + unc-119p::sid-1]	nervous system specific RNAi [94]	16.48 ± 0.31 12.47 ± 0.23	180 3	
TU3401 *ahr-1*			17.23 ± 0.32 13.03 ± 0.24	180	3	0.09 0.11
TU3311 con	uIs60[Punc119::YFP; Punc119::sid-1]	RNAi enhanced in the nervous system [94]	19.99 ± 0.44 15.87 ± 0.37	180	3
TU3311 *ahr-1*			17.50 ± 0.43 14.11 ± 0.37	180	3	0.001 0.003

Mean life- and healthspans +/- SEM of pooled data are shown. The L4440 plasmid was used as a negative control. Statistical analysis was performed between *ahr-1* and control RNAi using the Log-rank test. The number of worms is specified as N, while the number of replicates is specified as n.

### AHR-1 differentially regulates lifespan in response to potential modulators of its activity

**Figure 2:**
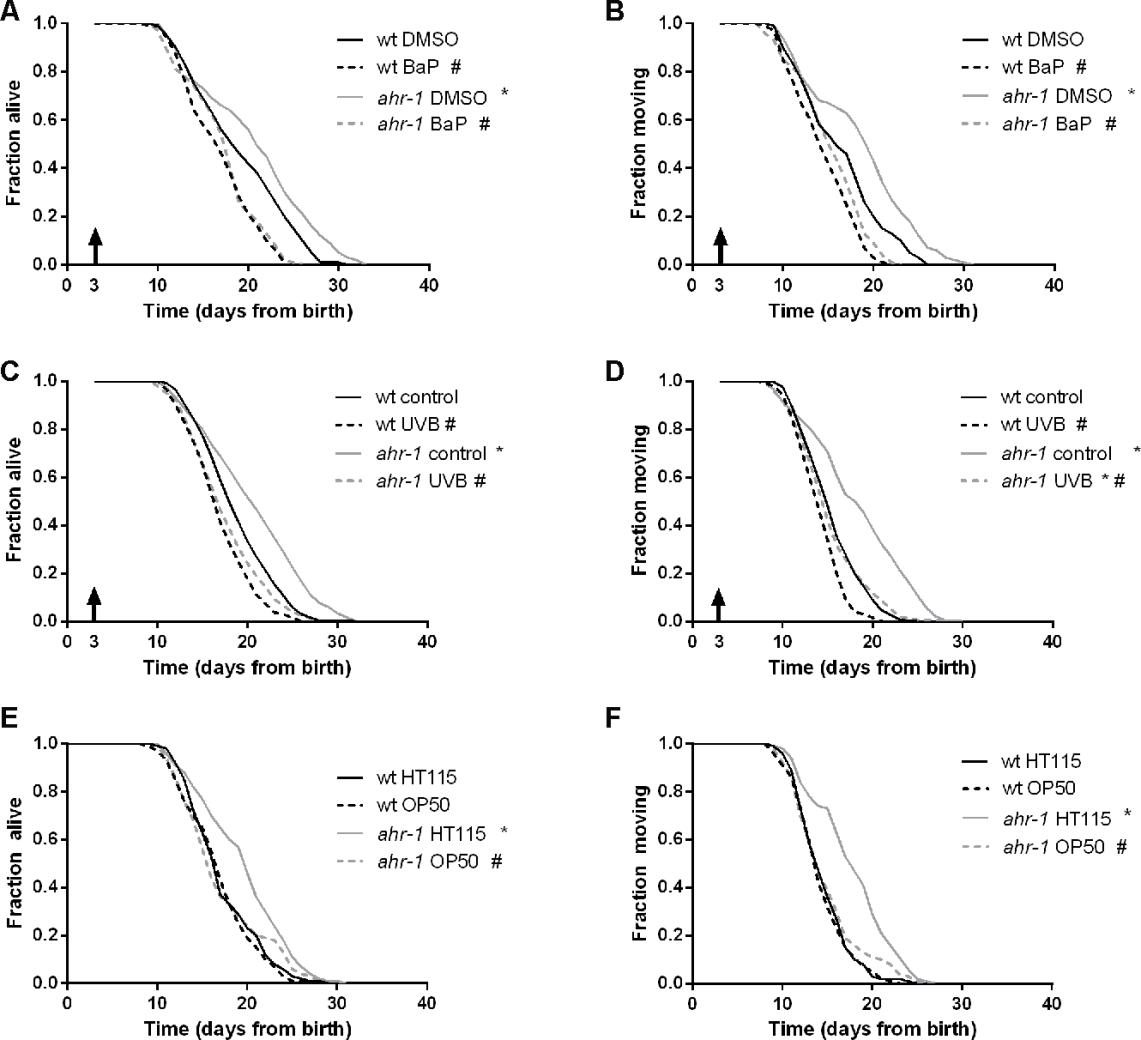
**AHR-1 displays evolutionarily conserved functions and affects aging in a diet-dependent manner.** (**A**, **B**) *ahr-1* is more sensitive to xenobiotic stress. Pooled lifespan/healthspan curves of 120 worms/condition in 2 independent replicates treated with either DMSO or 5 μM BaP from adulthood are shown. (**C**, **D**) *ahr-1* is more sensitive to UVB stress. Pooled lifespan/healthspan curves of 300 (wt control, *ahr-1* control), 180 (wt UVB) and 178 (*ahr-1* UVB) worms/condition in 3 independent replicates either left untreated or treated with 1200 J/m² UVB from adulthood are shown. (**E**, **F**) AHR-1 affects aging in a diet-dependent manner. Pooled lifespan/healthspan curves of 170 (wt OP50) and 180 (all other conditions) worms/condition in 3 independent replicates grown either on HT115 or OP50 are shown. (**A**–**F**) Statistical test: Log-Rank test, # significance *vs.* control/HT115, * significance *vs.* wt, p-value < 0.05.

In mammals, AhR exerts its functions primarily in response to ligands or modulators of its activity, such as exogenous substances (e.g. xenobiotics) or endogenous products of metabolism (e.g. microbiota-associated factors) [24]. The xenobiotic BaP exerts toxic effects in mammals through AhR-dependent *cyps* expression [25], and it can induce *cyps* expression also in *C. elegans* [34]. We observed that similar to mammals BaP significantly increased the expression of *cyp-35B1* and exerted toxic effects by affecting animals’ development in a dose-dependent manner. However, these effects were largely *ahr-1*-independent (Supplementary Figure 2A, 2B). Interestingly, we found that BaP treatment from adulthood, consistent with its toxic effects, significantly shortened animals’ health- and lifespan, with a more pronounced effect on *ahr-1* mutants, indicating a protective role of AHR-1 against BaP-curtailed longevity (Figure 2A, 2B). In mammals, UVB radiations activate AHR-1 through the formation of 6-formylindolo[3,2-b]carbazole (FICZ) [20, 21]. In *C. elegans* UVB radiation shortens lifespan, induces embryonic lethality and germline cells apoptosis in a dose-dependent manner [35, 36]. We showed that *ahr-1(ju145)* is more sensitive to UVB-induced embryonic lethality (Figure 1H). Accordingly, and in line with the notion that loss of AhR sensitizes mammalian cells to UVB-induced apoptosis loss [37], we found that UVB-induced germ cell apoptosis is significantly more increased in the *ahr-1* mutant’s germline compared to wild-type animals (Supplementary Figure 2C). UVB irradiation increases *Cyp1A1* expression in human keratinocytes in an AHR-dependent manner [20]. Interestingly, similar to the above results with BaP, although UVB tent to increase *cyp-35B1* expression in an *ahr-1*-independent manner (Supplementary Figure 2D), it decreased animals’ lifespan and motility with a significantly stronger effect on the *ahr-1(ju145)* compared to wild-type animals (Figure 2C, 2D). Our results indicate an evolutionarily conserved protective role of AhR in response to environmental AhR activators identified in vertebrates (BaP and UVB). Finally, as diet- and microbiota-associated factors modulate AhR activity in mammals, we took advantage of two common *Escherichia coli* strains used as a food source for *C. elegans*, HT115(DE3) and OP50(xu363). Strikingly, we found that the reduced expression of the *cyp-35B1*::GFP reporter (Supplementary Figure 3B), as well as the beneficial effects on lifespan, motility (Figure 2E, 2F), heat resistance and pharyngeal pumping (Supplementary Figure 3A, 3C, 3D), elicited in the *ahr-1* mutants fed HT115 are abolished when animals are fed OP50. Instead, the different bacteria neither affected the fertility in basal condition (Supplementary Figure 3E) nor the increased sensitivity to UVB (Supplementary Figure 3F, 3G) of the *ahr-1* mutant. This is consistent with our previous findings showing germline sensitivity to UVB-induced apoptosis does not correlate with lifespan outcomes [36]. The lipid droplet content, which did not change between wild-type and *ahr-1* mutants, was however similarly affected by the different bacteria (*not shown*). Results described so far indicate that known AhR modulators differentially affect *C. elegans* lifespan in an *ahr-1*-dependent manner but that classical detoxification related genes (*cyp*s) are possibly not the major targets of *C. elegans* AHR-1. To gain further insight into CeAhR plasticity of potential relevance for stress response and aging we turned to the results of a transcriptomic analysis recently carried out in the lab (Brinkmann et al. in preparation). A thorough analysis of the most differentially expressed genes between wild-type and *ahr-1(ju145)* revealed that many of them are known to be affected not only during *C. elegans* aging but also by dietary compounds (e.g. quercetin, resveratrol, bacteria), which modulate AhR activity in mammals (Tables 2 and 3). Interestingly, quantitative PCR analysis of some of these genes (*atf-2, K04H4.2, egl-46, T20F5.4, ptr-4, dyf-7, clec-209, C01B4.6, C01B4.7, F56A4.3*) confirmed their *ahr-1* dependency in basal conditions and supported a specific AhR-bacteria regulatory effect: their expression was generally not affected by UVB or BaP but their reduced expression in the *ahr-1(ju145)* mutants fed H115 bacteria, similar to *cyp-35B1*, was abolished in animal fed an OP50 diet (Supplementary Figure 4). Altogether, results shown so far indicate an evolutionarily conserved, protective role for AhR against BaP and UVB, and identify a new role for AhR in environmentally regulated aging with dietary bacteria as an important component in *ahr-1-*signaling-mediated longevity.

**Table 2:** List of the most robust over-expressed genes in *ahr-1* *vs.* wild-type.

**Gene Name**	**human ortholog**	**Molecular function**	**Modulators**	**logFC**	**adj. p-value**
*irld-35*		unknown	bacterial infection; temperature	1.968	0.0019
*clec-209*		carbohydrate binding	bacterial infection; aging; quercetin; heat shock	1.882	0.0182
*F56A4.2*		unknown	aging; bacterial infection; temperature	1.882	0.0182
*C01B4.6*	GALM	catalytic activity, isomerase activity, carbohydrate binding		1.671	9.3 * 10^-9^
*Y19D10A.16*	GALM	catalytic activity, isomerase activity, carbohydrate binding		1.671	9.3 * 10^-9^
*F56A4.3*	GSTP1	unknown	bacterial infection	1.660	0.0013
*srd-61*		unknown		1.614	0.0001
*srd-75*		unknown	bacterial infection	1.614	0.0001
*C01B4.7*		unknown	bacterial infection	1.605	0.0028
*Y19D10A.4*		transmembrane transporter activity	heat shock; bacterial infection	1.605	0.0028

Human orthologs were extracted using the BioMart tool (https://parasite.wormbase.org/biomart). Modulators of these genes with potential relevance for AHR-1 activity were selected from WormBase. For log fold change values (logFC) a base of 2 is used.

**Table 3:** List of the most robust under-expressed genes in *ahr-1* vs. wild-type.

**Gene Name**	**human ortholog**	**Molecular function**	**Modulators**	**logFC**	**adj. p-value**
*R02F11.1*		unknown	bacterial infection; bacterial diet	-2.308	0.0312
*hbl-1*	ZNF513
ZNF462	RNA polymerase II regulatory region sequence-specific DNA binding, RNA polymerase II transcription factor activity, sequence-specific DNA binding, transcription factor activity, metal ion binding	bacterial infection; quercetin; aging	-2.013	0.0171
*atf-2*		RNA polymerase II regulatory region DNA binding, transcriptional repressor activity, RNA polymerase II core promoter proximal region sequence-specific binding, transcription factor activity, protein binding	*ahr-1*; bacterial infection; quercetin; aging	-1.958	0.0227
*lpr-4*		unknown	bacterial infection; quercetin; bacterial diet; indole	-1.834	0.0227
*K04H4.2*		chitin binding	*ahr-1*; bacterial infection; bacterial diet	-1.831	0.0248
*egl-46*	INSM1 INSM2	RNA polymerase II transcription factor activity, sequence-specific DNA binding, RNA polymerase II transcription factor binding	bacterial infection; quercetin; aging	-1.823	0.0406
*T20F5.4*		unknown	bacterial infection; quercetin	-1.803	0.0400
*ptr-4*	PTCHD3	unknown	resveratrol; bacterial infection; quercetin; bacterial diet; aging	-1.766	0.0162
*lpr-5*		unknown	bacterial infection; bacterial diet; aging; quercetin; indole	-1.752	0.0227
*dyf-7*		protein self-association	quercetin; bacterial infection; temperature	-1.743	0.0227

Human orthologs were extracted using the BioMart tool (https://parasite.wormbase.org/biomart). Modulators of these genes with potential relevance for AHR-1 activity were selected from WormBase. For log fold change values (logFC) a base of 2 is used.

### Loss of *ahr-1* affects age-associated pathologies in a diet-dependent manner

**Figure 3:**
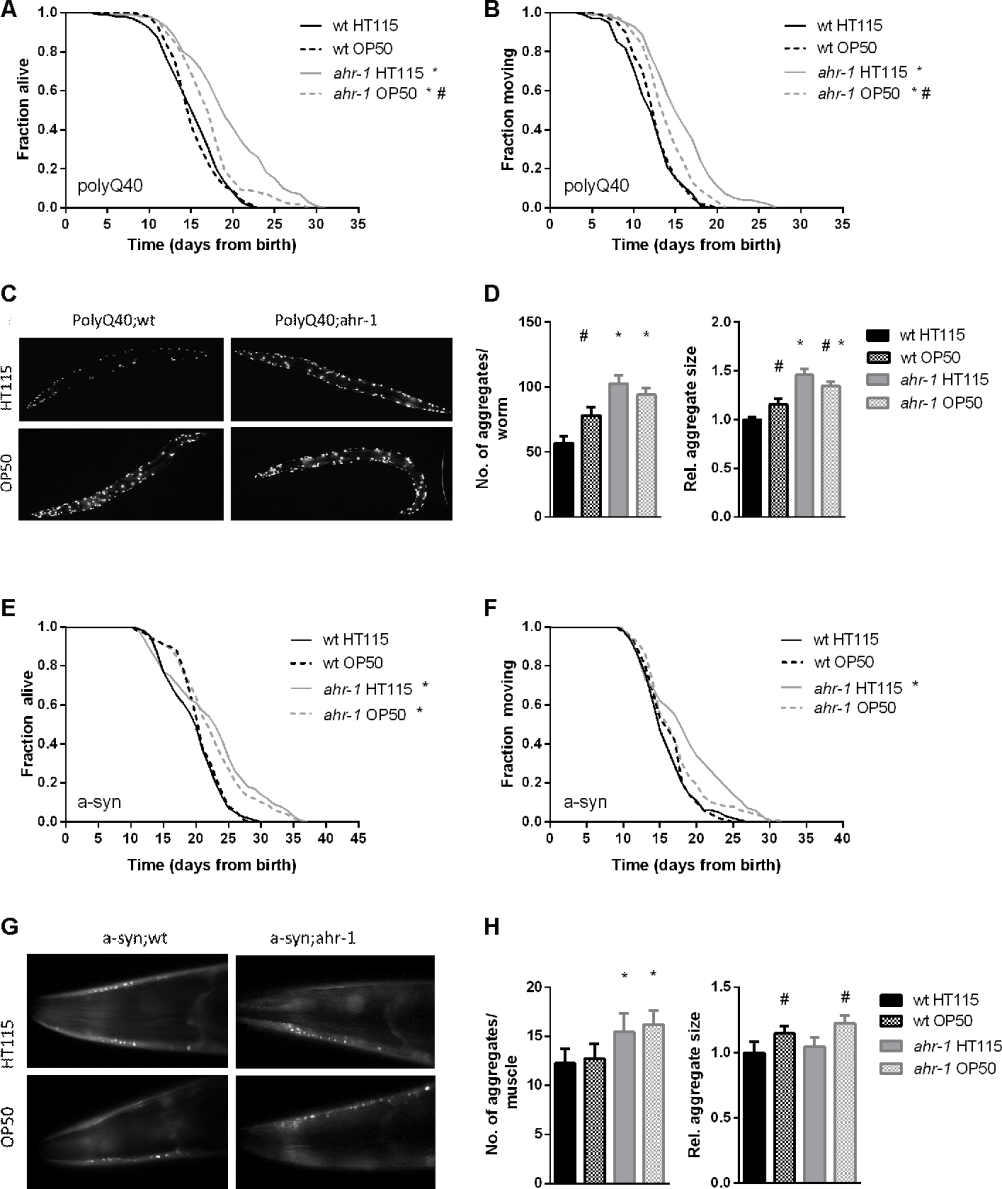
***ahr-1* mutants increase aggregation but extend lifespan in a diet-dependent manner.** (**A**, **B**) Kaplan Meier curves of polyQ;wt and polyQ;ahr-1 of 180 worms/condition in 3 independent experiments are shown. * p-value < 0.05 *vs.* polyQ;wt, # p-value < 0.05 *vs.* HT115, statistical test: Log-rank test. (**C**) Representative fluorescence images of 10-days old polyQ;wt and polyQ;ahr-1 on HT115 and OP50. (**D**) Quantification of aggregates in 10-days old polyQ;wt and polyQ;ahr-1. Mean + 95 % CI of pooled data from 34 (wt HT115), 29 (wt OP50), 26 (*ahr-1* HT115), and 35 (*ahr-1* OP50) worms in 3 independent replicates is shown. Statistical test: One-way ANOVA with Tukey’s multiple comparisons test, * p-value < 0.05 *vs.* polyQ;wt, ^#^ p-value < 0.05 *vs.* HT115. (**E**, **F**) Kaplan Meier curves of a-syn;wt and a-syn;ahr-1 of 120 worms/condition in 2 independent experiments are shown. * p-value < 0.05 *vs.* a-syn;wt, # p-value < 0.05 *vs.* HT115, statistical test: Log-rank test. (**G**) Representative fluorescence images of the head muscles of 7-days old a-syn;wt and a-syn;ahr-1 on HT115 and OP50. (**H**) Quantification of aggregates in 7- days old a-syn;wt and a-syn;ahr-1. Mean + 95 % CI of pooled data from 77 (wt HT115), 82 (wt OP50), 88 (*ahr-1* HT115), and 92 (*ahr-1* OP50) worms in 3 independent replicates is shown. Statistical test: One-way ANOVA with Tukey’s multiple comparisons test, * p-value < 0.05 *vs.* a-syn;wt, ^#^ p-value < 0.05 *vs.* HT115.

The critical role of AHR-1 in *C. elegans* neurons [14, 15, 17] and motility [8, 9] prompted us to investigate whether diet-dependent effects also influence age-related neuromuscular pathologies. A growing body of evidence indeed indicates microbiota can affect various aspects of *C. elegans’* health-span [38–42]. Notably, loss of *ahr-1* extended life- and health-span of two age-associated disease models, namely animals with muscle expression of aggregation-prone proteins polyglutamine (polyQ_40_) and α-synuclein (Figure 3), and these beneficial effects were significantly suppressed when animals were fed an OP50 diet instead of HT115 (Figure 3A, 3B, 3E, 3F). Despite being longer-lived, *ahr-1* mutants fed HT115 displayed a greater number and size of polyQ_40_ and α-synuclein aggregates, which is nonetheless in line with studies revealing no direct correlation between aggregates and toxic effects [43]. Moreover, the increase in aggregation was mainly regulated in a diet-independent manner (Figure 3C, 3D, 3G, 3H), indicating that the *ahr-1*-diet interaction effect on health-span is independent of its effect on protein aggregation. Somewhat surprisingly, when we investigated the effect of *ahr-1* deficiency in another pro-aggregation model - a *C. elegans* strain expressing a pan-neuronal human amyloid-beta (Aβ) peptide [44] - we found that contrary to the other models, it reduced animals’ life- and health-span, yet interestingly in a diet-dependent manner (Supplementary Figure 5A–5D).

**Figure 4:**
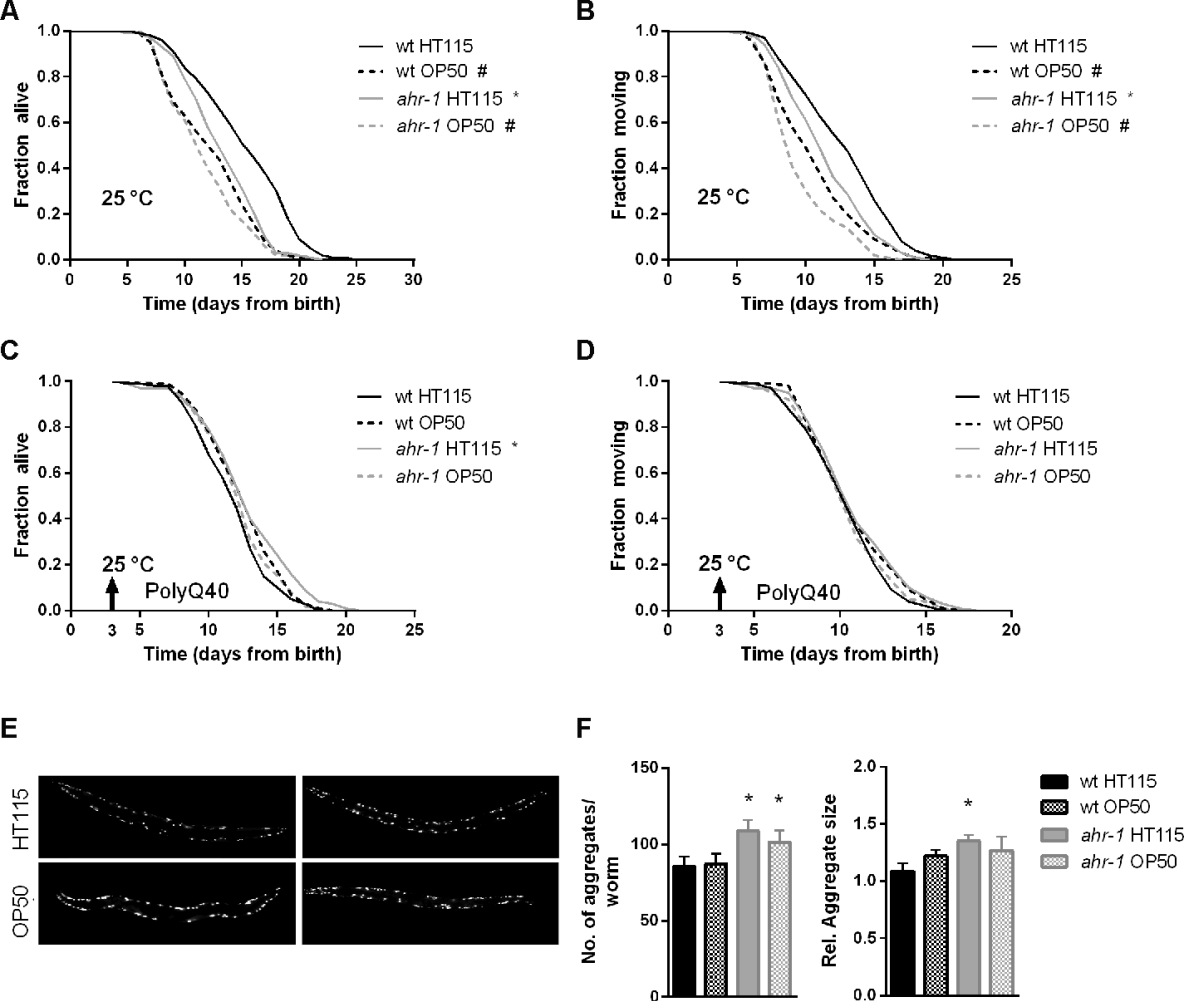
**At 25° C, *ahr-1(ju145)* is short-lived and loses its protection against the toxicity of polyQ_40_ aggregates.** (**A**, **B**) Kaplan Meier curves of wild-type and *ahr-1(ju145)* at 25° C. Pooled data of 180 worms/condition in 3 independent replicates are shown. * p-value < 0.05 *vs.* wt, # p-value < 0.05 *vs.* HT115, statistical test: Log-rank test. (**C**, **D**) Kaplan Meier curves of polyQ;wt and polyQ;ahr-1. Worms were grown at 25° C from day 3 (indicated by arrowhead). Pooled data of 150 worms/condition in 3 independent replicates are shown. * p-value < 0.05 *vs.* wt, # p-value < 0.05 *vs.* HT115, statistical test: Log-rank test. (**E**) Representative fluorescence images of 10-days old polyQ;wt and polyQ;ahr-1 grown at 25° C from day 3. (**F**) Quantification of aggregates in 10-days old polyQ;wt and polyQ;ahr-1 grown at 25° C from day 3. Mean + 95 % CI of pooled data from 21 (wt HT115), 19 (wt OP50), 20 (*ahr-1* HT115) and 19 (*ahr-1* OP50) worms/condition in 2 independent replicates are shown. * p-value < 0.05 *vs.* wt, statistical test: One-way ANOVA with Tukey’s multiple comparisons test.

Given that temperature may affect aging and associated pathologies depending on the bacterial diet and genetic background [38, 45] we next tested whether the healthy aging phenotype of *ahr-1* is also temperature-dependent. Despite the increased resistance to heat shock of *ahr-1(ju145)* mutants, and opposite to the lifespan at 20° C, *ahr-1*-depleted animals were short-lived on an HT115 diet at 25° C. Yet, again, this difference in the lifespan of wild-type and *ahr-1* was abolished on the OP50 diet, which *per se* already shortened the lifespan at 25° C of wild-type animals (Figure 4A, 4B). The higher temperature also prevented the beneficial diet-dependent effects of loss of *ahr-1* on life- and health-span in the polyQ strain (Figure 4C, 4D), while the number of polyQ aggregates increased according to the rise in temperature but still in a diet-independent manner (Figure 4E, 4F). Overall our data uncovered a new diet- and temperature-dependent effect of AhR in modulating aging and associated neuromuscular pathologies, which is nonetheless uncoupled from the age-dependent increase in protein aggregation.

### Bacterial tryptophan metabolism mediates the beneficial effect of the *ahr-1* mutant

**Figure 5:**
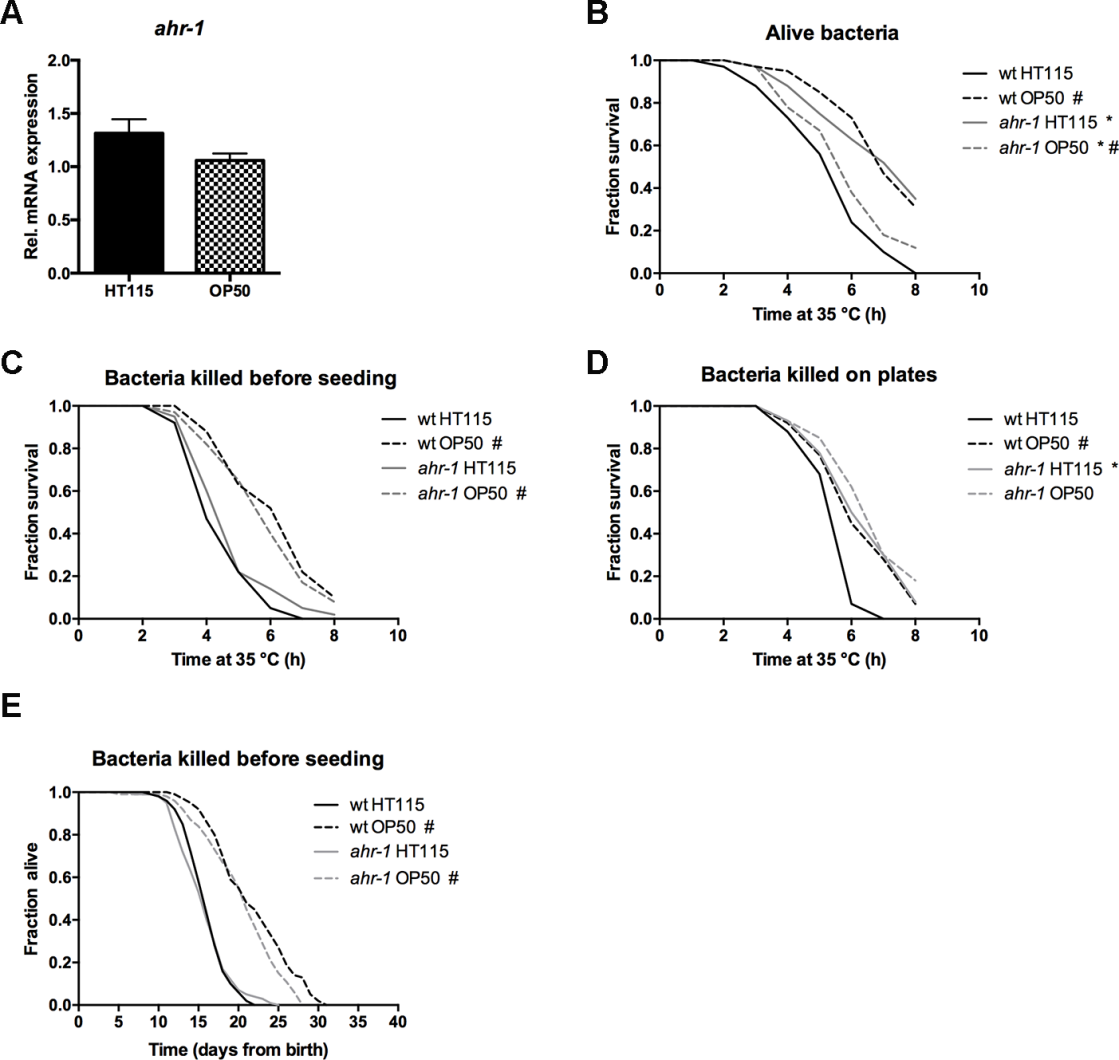
**Metabolically active bacteria are required for the differences in lifespan between wild-type and *ahr-1* on HT115.** (**A**) *ahr-1* mRNA expression in wild-type worms feeding on HT115 or OP50. Pooled data of 3 independent replicates are shown. No statistical significance was observed with the t-test. (**B**–**D**) Survival upon heat stress in 7-days old wild-type and *ahr-1(ju145)* feeding on either HT115 or OP50. Pooled data of 60 worms/condition in 3 independent experiments are shown. * p-value < 0.05 *vs.* wt, # p-value < 0.05 *vs.* HT115, statistical test: Log-rank test. (**B**) Alive bacteria were used as a food source. (**C**) Bacteria were killed by UVB irradiation before seeding to the NGM. (**D**) Bacteria had grown on the NGM for 2 days before being killed by UVB irradiation. (**E**) Kaplan Meier curves of wild-type and *ahr-1(ju145)* on UVB-killed bacteria. Pooled data of 120 (wt HT115, *ahr-1* HT115, *ahr-1* OP50) and 110 (wt OP50) worms in 2 independent experiments are shown. * p-value < 0.05 *vs.* wt, # p-value < 0.05 *vs.* HT115, statistical test: Log-rank test.

In search of the potential factor responsible for the diet-dependent differences, we first ruled out an effect of the different bacteria on the *ahr-1* expression (Figure 5A) and an effect of the different bacteria growing media (not shown). These results point to a specific role of bacteria components on the modulation of AHR-1-regulated processes. We thus asked whether metabolically active bacteria are required for the observed differences. To this end, we compared the heat-stress resistance of animals fed alive bacteria with that of animals fed bacteria either killed before seeding on plates or killed on the plates two days after seeding (thus allowing metabolites secretion). Very interestingly, killing bacteria before seeding completely abolished the differences between wild-type and *ahr-1* fed HT115 (Figure 5B, 5C), indicating that a factor produced by metabolically active HT115 bacteria may influence the AHR-1-mediated effects. In support of this possibility, the increased resistance to stress of *ahr-1* mutants observed on alive HT115 bacteria persisted when bacteria were killed after growing for two days on the feeding plates (Figure 5D). In line with the heat shock experiments, killing the bacteria before seeding also completely suppressed the increased lifespan of the *ahr-1* mutants on HT115 with no major effects on animals fed OP50 (Figure 5E). These data point towards HT115 secreted metabolites playing a role in *ahr-1*-mediated life- and health-span, likely via gut ingestion or neuronal sensing [38].

**Figure 6:**
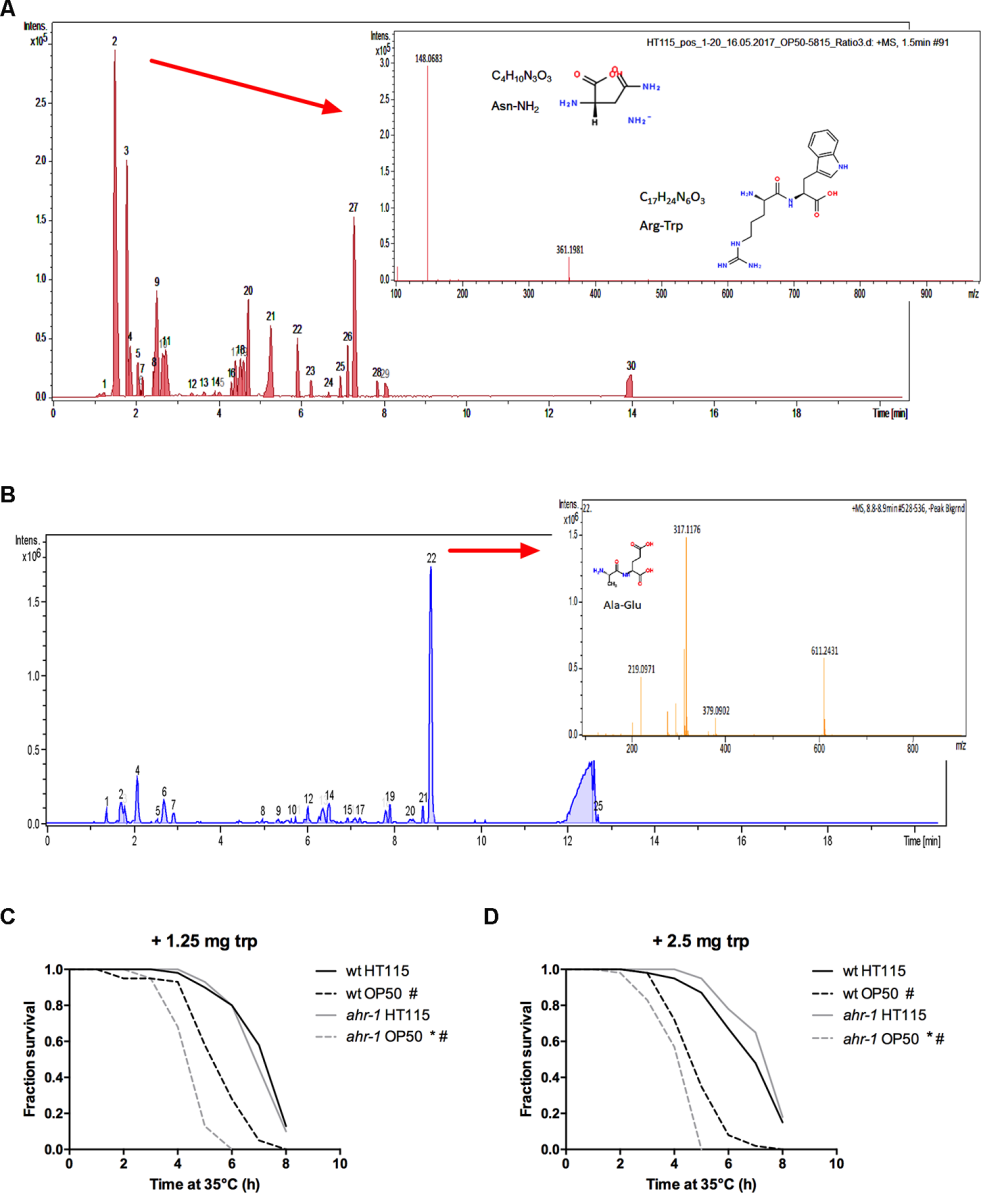
**Tryptophan supplementation abolishes the differences between wild-type and *ahr-1*.** (**A**) Positive ESI MS analysis. The HT115 BPC after the subtraction of the OP50 BPC is shown. Masses in peak 2 are shown as an inset. (**B**) Positive ESI-mass spectrum of OP50(L4440) medium after subtraction of the HT115(L4440) spectrum. Masses peak 22 are shown as an inset. m/z 219 is likely an Ala-Glu dipeptide. (**C**, **D**) Heat stress survival after tryptophan supplementation with indicated concentrations of tryptophan. Survival curves of 7-days old worms feeding on HT115 supplemented with tryptophan are shown. The curves show pooled data from or 40 worms/condition in 2 independent replicates (1.25 mg trp) or 60 worms/condition in 3 independent experiments (2.5 mg trp). * p-value < 0.05 *vs.* wt, # p-value < 0.05 *vs.* HT115, statistical test: Log-rank test.

A mass spectrometric analysis of the two bacteria supernatants revealed on the one hand that the subtracted spectrum between HT115 and OP50 showed three prominent peaks (Figure 6A) with m/z 361 likely being an arginine-tryptophan (Arg-Trp) dipeptide. On the other hand, the medium of OP50 was enriched in an alanine-glutamate (Ala-Glu) dipeptide (Figure 6B). In mammals, Trp and its metabolites (such as indole) modulate AhR activity [23, 46, 47] and in *C. elegans* Trp was shown to abolish some of the different phenotypes observed between alive HT115 and OP50 [48]. We found that supplementation of L-tryptophan completely abolished the heat-stress resistance of *ahr-1(ju145)* on HT115 - and partially suppressed the difference also between wild-type and *ahr-1(ju145)* on OP50 (Figure 6C, 6D). Overall, these data point towards a potential role of Trp metabolism in *ahr-1-*bacteria-regulated age-associated phenotypes.

## DISCUSSION

*C. elegans* AHR-1, similar to mammalian AhR, forms a heterodimer with the *C. elegans* Arnt homolog AHA-1 and binds to XRE. However, unlike its mammalian counterpart, CeAhR does not bind the classical AhR activators such as TCDD or β-naphthoflavone [12] indicating that the xenobiotic-metabolizing activity may not be an ancestral function of the receptor or it may have been lost during evolution in this nematode species. Regardless, to our knowledge, the effects of TCDD or other known AhR ligands or ligand-independent modulators have never been tested in *C. elegans* for their AhR-dependent effects, thus precluding to fully exploit the versatility of this model organism to unravel conserved or novel biological function for this environmentally relevant transcription factor.

Contradictory roles for AhR in the aging process have been identified (reviewed in [7]), but more recently, consistent pro-aging functions in health and lifespan were described across species [8, 9]. In this study, using *C. elegans* as a unique model organism to disclose novel potential functions of the AhR, we revealed a more complex role for this transcription factor in stress response and aging in basal conditions and in response to known vertebrate modulators of its activity. The differential effects on lifespan and gene expression we observed upon *ahr-1* genetic- or RNAi-mediated suppression (or upon different temperature), interestingly reveal the importance of fine-tuning AhR activity in a dose and/or tissue-dependent manner, which very nicely recapitulate the variety of phenotypic features described in mammals upon AhR dosages or tissue-specific depletion [7]. Although we assume *ahr-1* RNAi partially reduces AHR-1 expression and the *ahr-1(ju145)* is a loss-of-function allele, we did not in fact measure AHR-1 activity and therefore we cannot exclude that residual AHR-1 is more active or retain alternative functions.

To our surprise, we also found that besides increased resistance to heat-shock, lack of *ahr-1* did not confer resistance to other investigated insult, such as metabolic stress or radiation. We then specifically focus on the influence on stress response and aging of different classes of mammalian AhR modulators: environmental (xenobiotic and UVB) and dietary (microbiota) factors. These were shown to mainly induce AhR’s transcriptional activity in a ligand-dependent manner, but ligand-independent, as well as antagonistic functions, have been also suggested [11]. Our data showed that *C. elegans* *ahr-1(ju145)* mutants are more sensitive to the lifespan shortening effects of UVB and BaP, two classical activators of mammalian AhR, indicating a context-dependent role for AHR-1 in the aging process. Of note, *ahr-1* depletion also conferred sensitivity to UVB-reduced fertility, which we hypothesize to reflect increased sensitivity to germ cell apoptosis in irradiated animals as also found in human keratinocytes and mice [37, 49]. Indeed, loss of AHR-1 function increased apoptosis in basal condition and upon radiation indicating a conserved anti-apoptotic function of the AhR in response to UVB. Similar to the detrimental effect of UVB, exposure to BaP in mammals causes a variety of cancers as well as neurotoxicity [50, 51], and loss of AhR prevents BaP- and UVB-induced carcinogenicity in mice [25, 49]. In this study, we showed for the first time that BaP has detrimental effects also in an invertebrate, namely on lifespan, which is significantly worsened in the *C. elegans* *ahr-1* mutant. Along with the UVB data, these findings support a protective role of AHR-1 in response to classical mammalian activators. However, interestingly, both stressors induced *cyp-35B1* expression in an *ahr-1*-independent manner and *ahr-1* depletion inconsistently modulate the expression of other xenobiotic-response genes, especially Phase-I detoxification enzymes, while it seemed to have a more consistent effect on Phase-II enzymes such as *ugt-29* or *gst-4*. Although the expression of additional detoxification enzymes could be tested in *C. elegans*, our data point to an ancestral function of AhR different from its classical mammalian role in xenobiotic metabolism. This is further supported by our preliminary *in silico* analysis indicating BaP and the UVB-generated ligand FICZ are big and planar molecules that likely do not fit into the ligand binding pocket of the *C. elegans* AHR-1 (Brinkmann et al. *in preparation*). Instead, we speculate a protective AHR-1 role against the damaging effects induced by reactive oxygen species (ROS), which could be produced by UVB [52] or BaP [53, 54]. ROS could activate AHR-1 directly or through MAP kinases [55, 56] and the increased expression of the *gst-4* upon *ahr-1* depletion may indeed represent a basal activation of compensatory antioxidant response, such as the Nrf-2/*skn-1* redox transcription factor.

Most notably, we identified for the first time a critical role for AHR-1-bacterial diet interaction in regulating aging and associated phenotypes. As part of our initial attempt to identify environmental/dietary factors modulating aging in an AhR-dependent manner, we focused on the two most commonly strains used for *C. elegans* feeding, *E. coli* HT115 and *E. coli* OP50, which are known to differentially impact *C. elegans* aging-associated disease [38, 42]. We found that *ahr-1* mutants displayed an extended health- and lifespan on HT115 but not on the OP50 bacterial diet. It will be interesting to investigate the effects of other bacteria strains serving as food source and microbiota for *C. elegans* [57–59], as well as the effects of other dietary components (e.g. resveratrol, chalcones) known to extend *C. elegans* lifespan [60, 61] on AhR-control of the aging process. Loss of *ahr-1* also ameliorated health- and life-span in models of age-associated pathologies with aggregation-prone proteins expressed in the muscles in a diet-dependent manner. Yet, surprisingly, the beneficial effect did not correlate with the amount and size of the aggregates, indicating either that aggregation in this context has a beneficial effect, or that loss of *ahr-1* promotes health-span independently from mechanisms regulating proteotoxic aggregation. The effect of AHR-1 on aggregation-prone diseases may suggest novel targeted therapeutic options of relevance for different age-associated diseases. Interestingly, prototype AhR activators, such as TCDD and BaP, were shown to contribute to the development and progression of age-associated diseases (reviewed in [62, 63]) in different model systems [64] and a recent study showed increased AhR levels in serum brains of Alzheimer’s disease patients [65]. On the other hand, reduced α-synuclein levels were observed in the mouse ventral midbrain upon TCDD-induced AhR-dependent Parkin expression [66]. This contradictory effect of AhR on age-associated diseases is consistent with the impact of AhR on aging (reviewed in [7]) and likely represent the tip of the iceberg of a largely explored AhR function. Of note, our study indicates *C. elegans* represents a powerful model system to get insights into the role AhR in age-associated disorders.

We next sought to tackle the relevant bacterial component underlying the different bacteria effects on the *ahr-1-*dependent aging features. Some studies reported a beneficial effect of killed *E. coli* OP50 bacteria on *C. elegans* lifespan [67–69] but it has never been reported whether killed *E. coli* HT115 have a similar effect. Our data showed for the first time that the lifespan of nematodes fed UV-killed OP50 is significantly longer than that of those fed UV-killed HT115. However, while *ahr-1* mutants lived as long as wild-type on killed or alive OP50, killing HT115 completely abolished the differences between wild-type and *ahr-1*. We thus hypothesized a secreted bacterial metabolite from alive bacteria might mediate the diet-dependent changes in health-span. Little is known regarding these bacteria extruded metabolites [70, 71] and although the exact metabolite(s) responsible for the diet-dependent effects remains to be identified, our data point to the involvement of Trp metabolism. Our findings further support evolutionarily conserved functions of AHR-1 as Trp and its metabolites can influence AhR activity in mammals [23, 46, 47]. Trp and its metabolites such as indole and kynurenine have been also shown to modulate *C. elegans’* health- and lifespan. Trp supplementation increases heat-stress resistance and lifespan in *C. elegans* [72] and reduces the proteotoxicity in neurodegenerative disease models [43]. Instead, different Trp metabolites display opposite effects: Trp degradation through the Kynurenine pathway increases proteotoxicity in neurodegenerative disease models [43], while the tryptophan metabolite indole, from commensal bacteria, increases the lifespan of *C. elegans* through AHR-1 [73]. Our analysis on bacterial supernatant also indicated HT115 produces more alanine-glutamate dipeptide than OP50. Interestingly, alanine and glutamate were also shown to extend *C. elegans* lifespan [72]. Moreover, C. *elegans ahr-1* is required for cell fate specification of GABAergic neurons [17] and it was recently shown that HT115, differently from OP50, possess the enzyme (glutamate decarboxylase, GAD) necessary to convert glutamate into GABA, which is responsible to protect *C. elegans* neurons from degeneration [74]. Although the authors did not check whether OP50 and HT115 indeed produce different amounts of Glu, lack of GAD is expected, consistent with our data, to increase Glu production by OP50. Instead, different bacteria diets were shown to alter worms’ metabolic profile, with worms fed HT115 having more Glu than those fed an OP50 diet [39], likely to compensate for reduced Glu produced by the bacteria. Finally, compared to HT115 fed worms, OP50 were shown to confer sensitivity to oxidative stress possibly due to mitochondrial alteration and increased ROS production [39, 71, 75], thus further supporting a potential role for ROS in AHR-1 activation and/or downstream activities. It will be thus interesting to decipher the specific role of Trp, Glu and/or ROS metabolism in bacterial-AHR-1 regulation of the aging process. The primary site of action of the bacterial metabolite (e.g. intestine, sensory neurons) and the specific AhR-dependent molecular process responsible for the observed effects (e.g. immune response, redox reactions) are other attractive aspects to be elucidated.

In summary, we demonstrated that *C. elegans* *ahr-1* displays evolutionarily conserved functions, such as its protective activity against BaP and UVB, and identified a new direct link between *ahr-1*-regulated processes and bacterial diet as a key determinant of aging and associated pathologies, which may all rely on ancestral functions of the receptor related to responses to (oxidative) stress rather than xenobiotic metabolism. Since *C. elegans* does neither possess the liver (primarily involved in xenobiotic metabolism) nor an immune system, from an evolutionary point of view ROS metabolism may represent a primary line of defense against external toxicants and/or pathogens. Overall, our findings support a central role for AhR in the aging process in a context-dependent manner, thus expanding the already vast panel of activities played by the AhR in different pathophysiological conditions, and establish for the first time *C. elegans* as a powerful model organism to unravel new AhR-regulated processes in response to conserved modulators of its activity.

## MATERIAL AND METHODS

### *C. elegans* strains and cultivation

*C. elegans* strains used in this study are listed in Supplementary Table 2. We created the following strains for this study by crossing CZ2485 with different transgenic strains to obtain: NV33a: *ahr-1(ju145); cyp-35B1p::GFP + gcy-7p::GFP*, NV35a: *ahr-1(ju145); (pAF15)gst-4p::GFP::NLS*, NV38b: *ahr-1(ju145); unc-54p::Q40::YFP*, NV42a: *ahr-1(ju145); unc-54p::alpha-synuclein::YFP,* NV47a: *ahr-1(ju145*); *ugt-29*p::GFP. For maintenance, worms were kept synchronized by egg lay at 20° C on Nematode Growth Media (NGM) plates and fed with *E. coli* OP50 according to methods described in [76]. For the experiments, worms were synchronized on plates supplemented with 1 mM IPTG and *E. coli* HT115(L4440) or OP50(L4440) according to the condition of interest.

### Gene silencing by RNA-mediated interference (RNAi)

Gene silencing was achieved through feeding *E. coli* HT115(DE3) expressing plasmids with dsRNA against specific genes. RNAi feeding was applied continuously from birth to death.

### *E. coli* strains and growth

Bacteria were grown in LB medium at 37° C overnight. When using *E. coli* carrying vectors the LB medium was supplemented with 0.01 % of ampicillin and 0.0005 % of tetracycline. *E. coli* HT115(L4440), HT115(*ahr-1*), and OP50 were obtained from the Ahringer *C. elegans* RNAi library [77]. *E. coli* OP50*(xu363)* [41] was a gift from Shawn Xu.

### Heat stress survival

The resistance to heat stress was tested with 20 animals/condition per experiment at 35° C on 3 cm plates wrapped with parafilm in an incubator (Intrafors HT Multitron). The number of dead animals was scored hourly by gently touching the worms with a platinum wire and analysis was performed as described for the lifespan assay.

### Reproduction on heat stress, glucose, and BP

Animals were grown on control plates with alive bacteria until day 3 and then transferred to treatment or control plates for 24 hours. After a 24-hour treatment, 3 animals of each condition were transferred to fresh control plates for 4 hours to lay eggs and the number of eggs laid was counted. The number of progenies hatched from these eggs was counted 2 days afterward.

### Development

The development on the specific compound was explored by counting the number of eggs, which developed to gravid adults after 72, 96, and 120 hours as well as the number of worms that arrested their development and the number of eggs, which did not hatch.

### Glucose treatment

D-Glucose (Merck, 8342) was dissolved in ddH_2_O and supplemented to the NGM after autoclaving to reach concentrations of 2 % or 5 % and UVB killed *E. coli* HT115(L4440) were fed as a food source.

### 2,2′-dipyridyl (BP) treatment

The iron chelator 2,2′-dipyridyl (Carl Roth, 4153) was dissolved in ddH_2_O and supplemented to the NGM after autoclaving to reach concentrations of 50, 100, or 200 μM.

### UVB irradiation

Worms were exposed to ultraviolet radiation on bacteria-free NGM plates, using a Waldmann UV 236 B (UV6) lamp with an emission maximum of 320 nm. Irradiation times were 9 seconds, 27 seconds, and 53 seconds for 100 J/m², 300 J/m², and 600 J/m², respectively with a distance of 18 cm between lamp and plate.

### Reproduction after UVB treatment

3 days old worms were treated with UVB and the effect of irradiation on germ-cells in different stages (oogenic stage, meiotic stage, and mitotic stage) was investigated, as described in [78]. Briefly, the number and viability of eggs laid between 1 – 8 hours (oogenic stage), 8 – 24 hours (meiotic stage), and 24 – 32 hours (mitotic stage) after the irradiation were analyzed.

### Lifespan

The lifespan analysis was started from a synchronized population of worms, which was transferred to fresh NGM plates daily during the fertile period. After the fertile phase, the animals were transferred every alternate day. Dead, alive, and censored animals were scored. Animals with internal hatching (bags), an exploded vulva, or which died desiccated on the wall were censored. Survival analysis was performed in OASIS [79] or OASIS 2 [80] using the Kaplan Meier estimator. A log-rank test between pooled populations of animals was used for the evaluation of statistical significance. The p-values were corrected for multiple comparisons using the Bonferroni method.

### Movement/healthspan

The movement was set as a parameter for healthy aging, and the phase of the active movement is referred to as healthspan. It was assessed in the populations used for the lifespan assay. Animals, which were either crawling spontaneously or after a manual stimulus, were considered as moving while dead animals or animals without crawling behavior were considered as not moving. Statistical analysis was done as described for lifespan.

### Benzo(a)pyrene (BaP) treatment

Benzo(a)pyrene (Sigma Aldrich, B1760) was dissolved in DMSO (Carl Roth, 4720) in concentrations 1000 times higher than the desired concentration in the NGM. After autoclaving the NGM, BaP, or DMSO were added to the media. We used final concentrations of 0.1 % DMSO or 0.1 μM, 1 μM, 5 μM, or 10 μM BaP. For development assays, worms were treated from eggs, while they were treated from the first day of adulthood for lifespan and healthspan assays.

### Quantification of polyQ aggregates

PolyQ_40_ aggregates were visualized by fluorescence microscopy (100x magnification) in worms anesthetized with 15 mM sodium azide (Sigma, S2002). The number and the size of the aggregates were quantified in Fiji [81]. To assess the number of aggregates, images were stitched using the Fiji pairwise stitching plugin [82] to create whole worms. The average size of the aggregates was instead measured in the non-stitched images. The number and the size of aggregates were counted using the plugin “Analyze Particles”.

### Quantification of α-synuclein aggregates

α-synuclein aggregates in the head muscles of 7-days old worms were visualized by fluorescence microscopy (400x magnification) in worms anesthetized with 15 mM sodium azide (Sigma, S2002). Pictures were segmented using Ilastik (version 1.3.0) (available on https://www.ilastik.org/) [83]. The segmented pictures were used to analyze the number and size of the aggregates in Fiji [81] using the plugin “Analyze Particles”.

### Assessment of age-associated features at 25° C

Lifespan, movement, and polyQ aggregation analysis were performed as described above. PolyQ-expressing worms were kept at 20° C until reaching the L4 stage and were afterward kept at 25° C for the rest of their lifespan.

### Killing bacteria before seeding

When seeding killed *E. coli* onto the NGM, the bacteria were pelleted (10 min at 4000 rpm), the supernatant was removed, and the pellet was suspended in S-basal to a final concentration of OD_595_ = 3.6. Then the bacteria suspension was irradiated with a UVB lamp (Waldmann UV 236 B) for 1 hour to kill the bacteria. The suspension of the dead bacteria was again pelleted and re-suspended in fresh S-basal. Killed bacteria were seeded to NGM plates in a concentration of OD_595_ = 3.6 and let dry at room temperature overnight.

### Killing bacteria on plates

Bacteria (OD_595_ = 0.9) were seeded to the NGM plates and let grow for 2 days at room temperature. The bacterial lawn was then killed by exposure to UVB light (Waldmann UV 236 B) for 45 min.

### Tryptophan supplementation

The concentration of tryptophan and the supplementation procedure was taken from [48] with minor changes. L-tryptophan (Carl Roth 4858.2) was dissolved in water at a concentration of 12.5 mg/ml and incubated shaking at 30° C for 45 min. Afterward, the solution was filtered (pore size: 0.22 μm, Carl Roth P666.1). For 7 ml of NGM 200 μl of 12.5 mg/ml tryptophan was spotted on the bacterial lawn of an NGM plate, which was kept at room temperature overnight. After the supplementation, the dish was kept at room temperature for another day.

### Mass spectrometry

Liquid NGM was prepared like solid NGM but without agar to prevent solidification. NGM was poured into petri dishes (7 ml NGM/ 6 cm petri dish) and seeded with bacteria (200 μl, OD_595_ = 0.9) or LB-medium (control). The medium was incubated for two days at room temperature to allow the bacteria to grow. Then, the bacteria were removed by centrifugation (4500 rpm, 10 min) and the medium was filtered (22 μm filter, Carl Roth P666.1) before using it for mass spectrometry analysis. Electrospray ionization mass spectrometry (ESI-MS) was used. For the LC-MS measurements, the liquid NGM samples were diluted 1:20 with methanol before the injection of 10 μl sample volumes. Triterpenes were separated on a Dionex HPG 3200 HPLC system (Thermo Scientific) equipped with a 150 x 2.1 mm, 2.7 μm, C18-CSH column (Waters) with a binary gradient system. Mobile phase A consisted of water + 0.1 % formic acid (FA), and mobile phase B consisted of methanol + 0.1 % FA. The mobile phase gradient was as follows: Starting conditions were 5 % mobile phase B, increased to 95 % B within 10 min, the plateau was held for 4 min, and the system was returned to starting conditions within 1 min and held for another 4.5 min. The flow rate was 0.5 mL/min. The MS and MS/MS analysis were performed with a quadrupole-time-of-flight instrument (maXis 4G, Bruker Daltonics, Bremen, Germany) equipped with an ESI source. The device was operated in positive-ion and negative-ion mode and the operating conditions were as follows: dry gas (nitrogen): 8.0 L/min, dry heater: 220° C, nebulizer pressure: 1.8 bar, capillary voltage: 4500 V. Data analysis were performed using the software data analysis 4.2 and Metabolite detect 2.1 (Bruker daltonics, Bremen, Germany).

### Microarray

For the analysis of gene expression by microarray, samples from 5 independent replicates with approximately 1000 3-days old worms grown on NGM plates containing 0.1 % DMSO were washed with S-basal 3 times and then collected in H_2_O. The mRNA was extracted using the RNeasy-/Shredder Kit (Qiagen, 74104 and 79654). The samples were further processed and applied to an Affymetrix Chip. The microarray raw data in the format of CEL were analyzed using the software R (version 3.4.2) and Bioconductor [84]. Background correction, normalization, and expression calculation of all the array were performed with the oligo package [85] and the RMA method. Then, quality control of the array was run using the package arrayQualityMetrics_3.34.0 [86].

### Assessment of mRNA expression by RTqPCR

Samples from 3 independent replicates with approximately 1000 3-days old worms per condition were collected and RNA was extracted. After washing and elution steps the RNA content was quantified by spectrophotometry, and 1 - 2 μg of RNA was used for the cDNA synthesis (Omniscript RT Kit (Qiagen, 205111). Primers were designed using NCBI Primer BLAST (https://www.ncbi.nlm.nih.gov/tools/primer-blast/) [87]. Each primer pair was designed to span an exon-exon junction. Primer pairs and their features are listed in Supplementary Table 3. For the Real-time qPCR, the cDNA was diluted 1:20 in 10 mM TRIS (pH 8.0). For the reaction, the GoTaq® qPCR kit (Promega, A6001) was used. The samples were run in a MyiQ2 cycler (BioRad), and the expression of each sample was measured in duplicate on the same multi-well plate. The expression was calculated relative to the reference genes *act-1* and *cdc-42* using the iQ5 software. All data collected were enabled for gene study according to the BioRad user instructions, and the expression was calculated using the normalized expression (ddC_T_). The efficiency of each primer pair reaction was added for correct quantification of the normalized expression. The efficiency was assessed with 1:20, 1:100, 1:500, and 1:2500 dilutions of the cDNA. From normalized expression values, the fold-change compared to wild-type was calculated for each replicate.

### Statistical analysis

If not stated, statistical analysis was performed in GraphPad Prism 6. For life-/healthspan assays, statistical analysis was done using OASIS [79, 80].

## Supplementary Material

Supplementary Materials and Methods

Supplementary Figures

Supplementary Tables
