# Supplementary material for: Dietary and environmental factors have opposite AhR-dependent effects on C. elegans healthspan
Source: Aging (Albany NY). 2020 Dec 13;13(1):104–33. doi: 10.18632/aging.202316 (PMC7835051; doi:10.18632/aging.202316)
Supplement: Supplementary Materials and Methods [file aging-13-202316-s001.pdf]

SUPPLEMENTARY MATERIALS

MATERIALS AND METHODS

Transformation of *E. coli* OP50(xu363)

For this study, we created OP50(L4440) by PEG transformation of OP50(xu363) according to methods described previously with adjustments [1]. 100 µl of OP50(xu363) were transformed with 100 pg of plasmids isolated from the HT115(DE3) strains (Ahringer Library). The QIAprep Spin Miniprep Kit (Qiagen, 27104) was used for plasmid isolation.

Crossing CZ2485 with transgenic strains

CZ2485 males were generated by treating hermaphrodite L4 larvae with heat stress (4 h at 30° C). 10 adult CZ2485 males were then left with one hermaphrodite L3/L4 larvae for mating (P0 generation). Single worms of the F1 and F2 generations of this cross were checked for the *ju145* allele by PCR (ahr-1F CGGAAAGTTGATGTCTCTAC, ahr-1R TGCTGACT AGACGATATACC) followed by a restriction with *AlwI* (New England Biolabs, R0513S), which cuts the PCR fragment at the position of the *ju145* point mutation. Single worm PCR was performed according to standard protocols [2].

Quantification of transgene expression

The expression of fluorescently-tagged genes was investigated in adult worms (first day of adulthood) by using fluorescence microscopy (ZEISS Imager M2 with an Axiocam MRm camera). For this, worms expressing fluorescently tagged genes were paralyzed with 15 mM NaN_3 and pictures were taken with identical exposure times and settings. The pictures were then analyzed in either Image J, Fiji [3] or Cellprofiler. Depending on the distribution of the expression, the fluorescence was either measured in a defined area of the region of interest or the whole worm. Statistical analysis was performed with the pooled data.

Measurement of *cyp-35B1p::GFP* intensity in response to BaP

3-days old worms were treated with BaP or UVB for 18 hours. The relative intensity of *cyp-35B1p::GFP* was then visualized by fluorescence microscopy (100x magnification) and analyzed using Fiji [3]. The integrated density was chosen as a parameter for the expression. The quantified intensities were normalized to the mean of untreated wild-type in each experiment. Statistical analysis was performed with the pooled data.

UVB-induced apoptosis

To investigate UVB-induced apoptosis, L4 larvae were treated with 600 J/m² UVB and the apoptotic corpses were counted 24 h post-irradiation in the gonad loop region. The apoptotic corpses were identified based on their shape.

Fertility

The number of eggs and progeny of animals were investigated during the main fertile period. Two days after synchronization single L4 larvae were transferred to NGM plates and from then transferred to fresh NGM plates every 24 hours until the 8th day after hatching. The number of eggs laid during this period was counted. Two days later the progeny hatched on each day were counted.

Pharyngeal pumping using the NemaMetrix ScreenChip system

To measure the pharyngeal pumping rate with the NemaMetrix ScreenChip, worms were washed off the plates with S-basal and collected in a reaction tube. Then, the worms were washed twice with S-basal and twice with 10 mM serotonin (Sigma Aldrich, 14927) and incubated in 10 mM serotonin for 30 min. Worms were loaded on the ScreenChip SC40 with a syringe (0.01 ml – 1 ml). The EPG of single worms was recorded for a duration of approx. 2 minutes. Only worms which showed pumping activity were recorded, while those with no pumping activity were not considered. The following NemAcquire-2.1 and NemAnalysis-0.2 software were used for analysis (https://nemametrix.com/products/software/).

Assessment of mRNA expression by RTqPCR

Samples from 3 independent replicates with approximately 1000 3-days old worms per condition were collected and RNA was extracted. After washing and elution steps the RNA content was quantified by spectrophotometry, and 1 - 2 µg of RNA was used for the cDNA synthesis (Omniscript RT Kit (Qiagen, 205111). Primers were designed using NCBI Primer BLAST (https://www.ncbi.nlm.nih.gov/tools/primer-blast/) [4]. Each primer pair was designed to span an exon-exon junction. Primer pairs and their features are listed in Supplementary Table 3. For the Real-time qPCR, the cDNA was diluted 1:20 in 10 mM TRIS (pH 8.0). For the reaction, the GoTaq® qPCR kit (Promega, A6001) was used. The samples were run in a MyiQ2 cycler (BioRad), and the expression of each sample was

measured in duplicate on the same multi-well plate. The expression was calculated relative to the reference genes *act-1* and *cdc-42* using the iQ5 software. All data collected were enabled for gene study according to the BioRad user instructions, and the expression was calculated using the normalized expression (ddC$_T$). The efficiency of each primer pair reaction was added for correct quantification of the normalized expression. The efficiency was assessed with 1:20, 1:100, 1:500, and 1:2500 dilutions of the cDNA. From normalized expression values, the fold-change compared to wild-type was calculated for each replicate.

REFERENCES

1. Chung CT, Miller RH. A rapid and convenient method for the preparation and storage of competent bacterial cells. Nucleic Acids Res. 1988; 16:3580. https://doi.org/10.1093/nar/16.8.3580 PMID:3287331

2. He F. Single Worm PCR. Bio Protoc. 2011; Bio101:e60. https://doi.org/10.21769/BioProtoc.60

3. Schindelin J, Arganda-Carreras I, Frise E, Kaynig V, Longair M, Pietzsch T, Preibisch S, Rueden C, Saalfeld S, Schmid B, Tinevez JY, White DJ, Hartenstein V, et al. Fiji: an open-source platform for biological-image analysis. Nat Methods. 2012; 9:676–82. https://doi.org/10.1038/nmeth.2019 PMID:22743772

4. Ye J, Coulouris G, Zaretskaya I, Cutcutache I, Rozen S, Madden TL. Primer-BLAST: a tool to design target-specific primers for polymerase chain reaction. BMC Bioinformatics. 2012; 13:134. https://doi.org/10.1186/1471-2105-13-134 PMID:22708584
