# Supplementary material for: Dietary and environmental factors have opposite AhR-dependent effects on C. elegans healthspan
Source: Aging (Albany NY). 2020 Dec 13;13(1):104–33. doi: 10.18632/aging.202316 (PMC7835051; doi:10.18632/aging.202316)
Supplement: Supplementary Figures [file aging-13-202316-s002.pdf]

SUPPLEMENTARY FIGURES

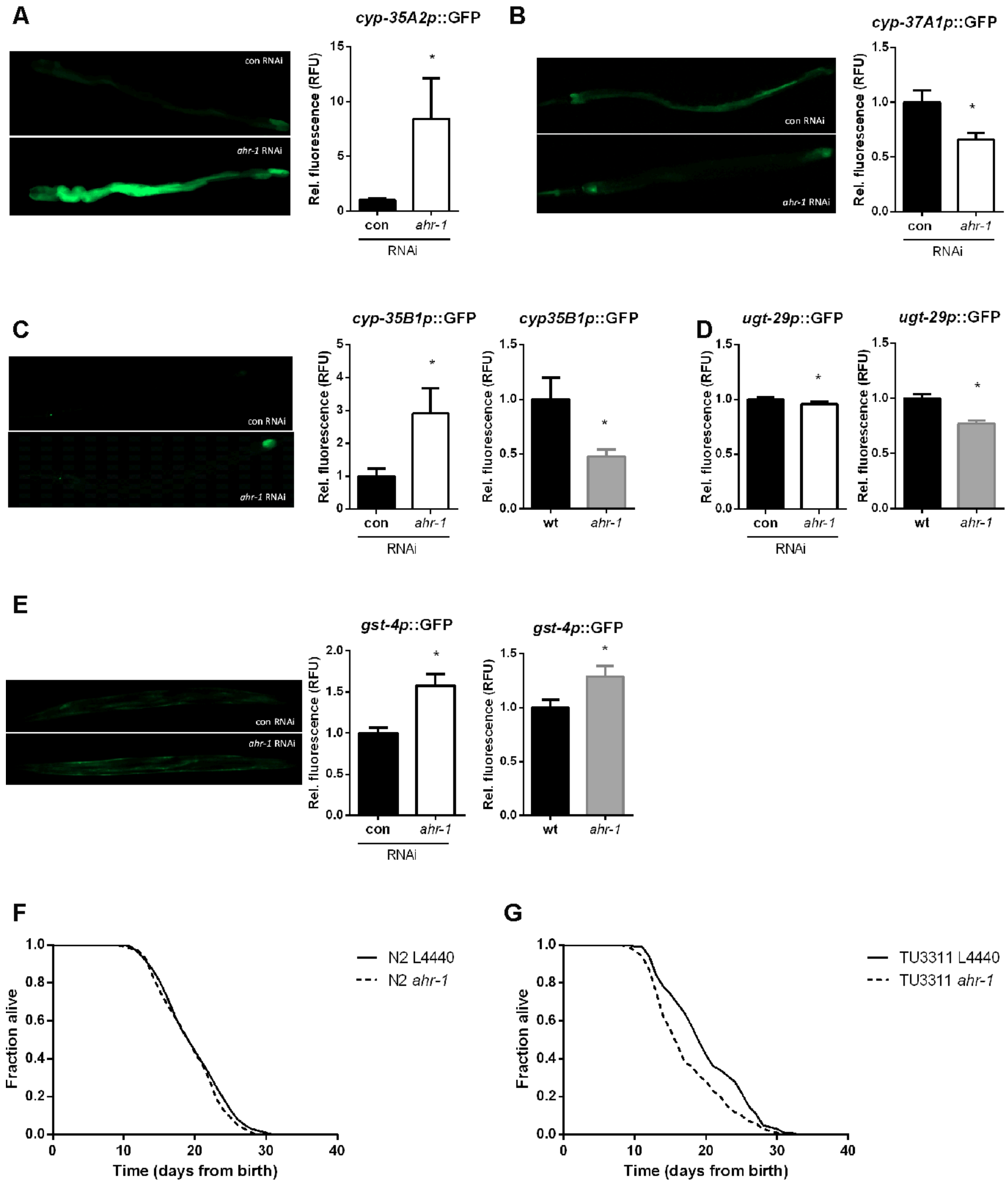

Supplementary Figure 1. AHR-1 regulates Phase I and Phase II enzyme genes. (**A**) Quantification of cyp-35A2p::GFP expression in worms treated with control or *ahr-1* RNAi. Mean + 95 % CI of pooled data from 3 independent experiments with 43 worms are shown. (**B**) Quantification of cyp-37A1p::GFP expression in worms treated with control or *ahr-1* RNAi. Mean + 95 % CI of pooled data from 2 independent experiments with 38 (con RNAi) and 26 *(ahr-1* RNAi) worms are shown. (**C**) Quantification of cyp-35B1p::GFP expression in worms treated with control or *ahr-1* RNAi (left panel) or worms with either wild-type *ahr-1* or the *ju145* allele (right panel). Mean + 95 % CI of pooled data from 60 (con RNAi), 40 (*ahr-1* RNAi), 52 (wt), and 63 (ahr-1) worms in 3 independent experiments are shown. (**D**) Quantification of ugt 29p::GFP expression in *ahr-1*-depleted worms. Mean + 95 % CI of pooled data from 3 independent experiments are shown. Number of worms 44 (con RNAi), 36 (ahr-1 RNAi) [left panel], 34 (wt), 40 (ahr-1) [right panel]. (**E**) Quantification of gst-4p::GFP expression in worms treated with control or *ahr-1* RNAi (left panel) or worms with either wild-type *ahr-1* or the *ju145* allele (right panel). Mean + 95 % CI of pooled data from 27 (con RNAi), 29 (ahr-1 RNAi), 69 (wt), and 66 (ahr-1) worms in 2 or 4 independent experiments are shown. (**F**) ahr-1 depletion via RNAi does not affect the lifespan of N2. Kaplan Meier curves of control (L4440) or ahr-1 RNAi treated worms. Pooled data of 280 (N2 L4440) and 264 (N2 ahr-1) worms/condition in 5 independent replicates are shown. * p-value < 0.05 vs. wt, statistical test: Log-rank test. (**G**) ahr-1 depletion via RNAi shortens the lifespan in a strain with enhanced RNAi in the nervous system (TU3311). Kaplan Meier curves of control (L4440) or ahr-1 RNAi treated worms. Pooled data of 180 worms/condition in 3 independent replicates are shown. * p-value < 0.05 vs. wt, statistical test: Log-rank test.

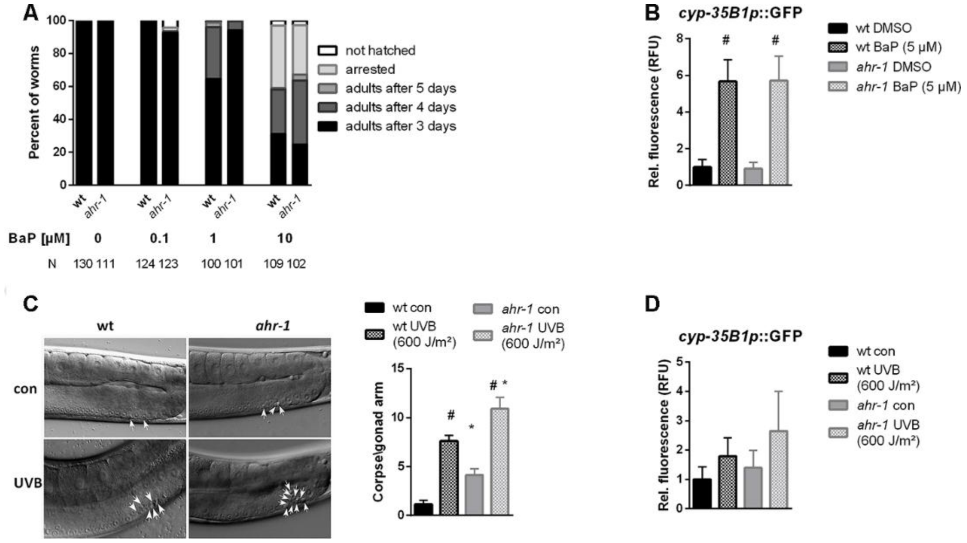

Supplementary Figure 2. Responses of *C. elegans* to known mammalian AhR activators. (**A**) Development of wild-type and *ahr-1* on indicated doses of BaP. Pooled data of 3 independent experiments are shown, the number of individuals is presented as N. Statistical test: 2-Way ANOVA with Tukey's multiple comparisons test, no statistical differences were observed. (**B**) cyp-35B1p::GFP induction in response to 5 µM of BaP. Mean + 95 % CI of pooled data of 58 (wt DMSO), 51 (wt BaP), 53 (ahr-1 DMSO), and 65 (ahr-1 BaP) in 3 independent experiments are shown. * p-value < 0.05 vs. wt, # p-value < 0.05 vs. DMSO, statistical test: One-way ANOVA with Tukey's multiple comparisons test. (**C**) UVB irradiation and *ahr-1* loss of function induce apoptosis. Mean + 95 % CI of pooled data of 39 (wt con, ahr-1 UVB) and 38 (wt UVB, ahr-1 con) worms in 3 independent experiments are shown. Statistical test: 2-Way ANOVA with Tukey's multiple comparisons test, * p-value < 0.05 vs. wt, # p-value < 0.05 vs. con. (**D**) cyp-35B1p::GFP induction in response to UVB irradiation. Mean + 95 % CI of pooled data of 34 (wt con), 37 (wt UVB), 28 (ahr-1 con), and 35 (ahr-1 UVB) worms in 2 independent experiments are shown. * p-value < 0.05 vs. wt, # p-value < 0.05 vs. control, statistical test: One-way ANOVA with Tukey's multiple comparisons test.

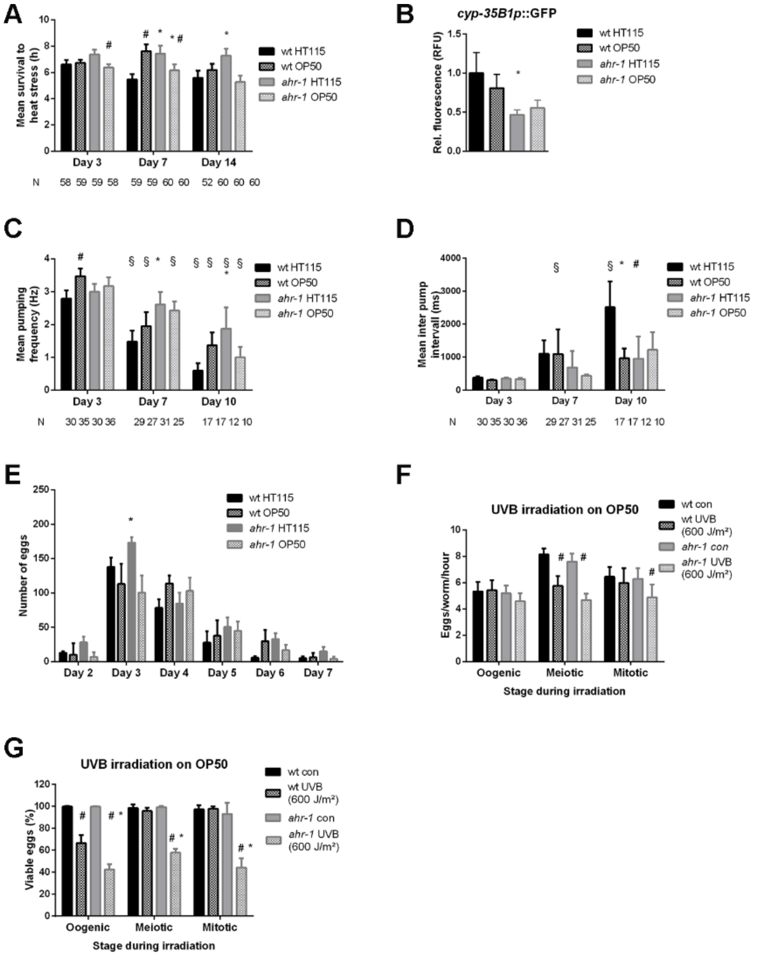

Supplementary Figure 3. Aging is influenced by the bacterial diet in the *ahr-1* mutant. (**A**) Heat shock resistance of wild-type and *ahr-1* feeding on HT115 or OP50. Mean survival times + SEM of 3 independent experiments are shown, the number of individuals is presented as N. Statistical test: Two-way ANOVA with Tukey's multiple comparisons test. * p-value < 0.05 vs. wt, # p-value < 0.05 vs. HT115, (**B**) cyp-35B1p::GFP expression of worms feeding on HT115 or OP50. Mean + 95 % CI of pooled data of 37 (wt HT115), 27 (wt OP50), 44 (ahr-1 HT115), and 19 (ahr-1 OP50) in 2 independent experiments are shown. * p-value < 0.05 vs. wt, # p-value < 0.05 vs. HT115, statistical test: One-way ANOVA with Tukey's multiple comparisons test. (**C**, **D**) Pumping frequencies and inter pump intervals of pooled data from 3 independent experiments are shown as mean + 95 % CI, the number of individuals is presented as N. * p-value < 0.05 vs. wt, # p-value < 0.05 vs. HT115, § p-value < 0.05 vs. day 3, statistical test: Two-way ANOVA with Tukey's multiple comparisons test. (**E**) Comparison of the fertility of worms feeding on HT115 or OP50. Means (+SEM) of 3 independent replicates are shown. (**F**, **G**) Fertility of worms feeding on OP50 in response to a dose of 600 J/m²of UVB. Means (+SEM) of 3 independent replicates are shown.

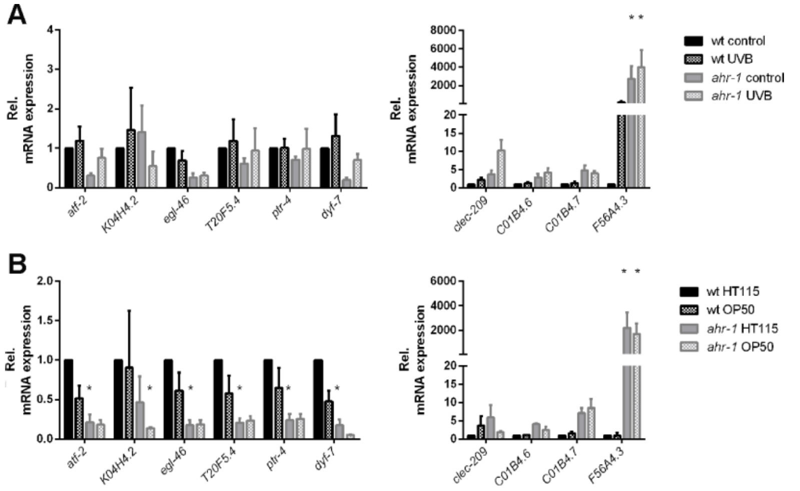

Supplementary Figure 4. Gene expression changes in response to AhR modulators. Expression of the strongest up- and down-regulated genes in *ahr-1* vs. wild-type in response to (**A**) 1200 J/m² UVB, and (**B**) bacteria. Pooled data from 3 independent replicates are shown. Statistical test: 2-Way ANOVA with Tukey's multiple comparisons test, * significance vs. wt, # significance vs. control.

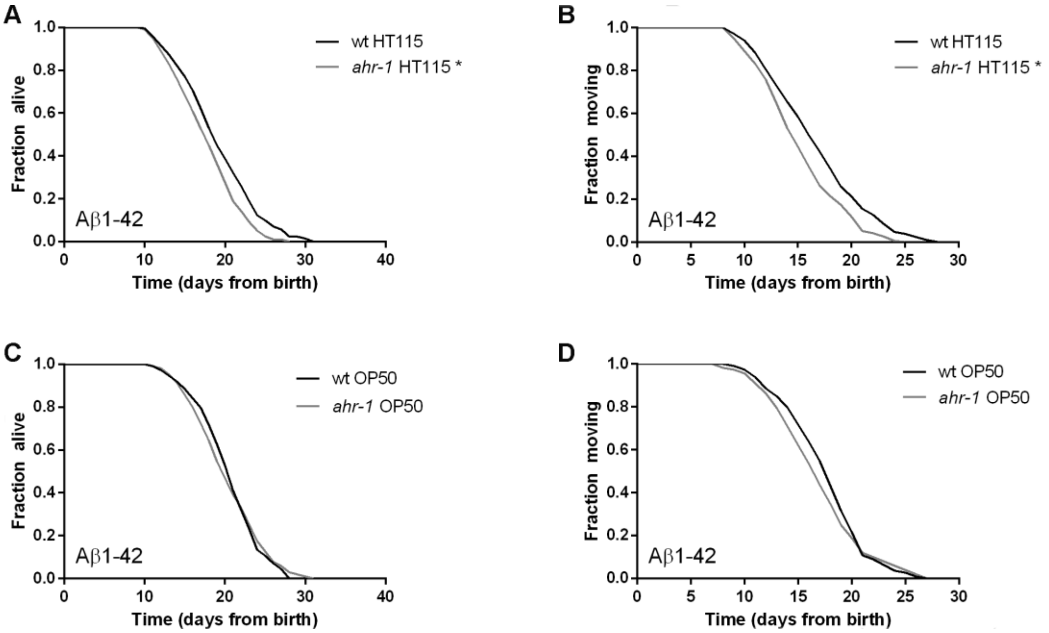

Supplementary Figure 5. AHR-1 does not influence aging in an Alzheimer's disease model. (**A**, **B**) Kaplan Meier curves of Abeta;wt and Abeta;ahr-1 grown on HT115. Pooled data of 240 (wt) and 236 (*ahr-1*) worms/condition in 4 independent replicates are shown. * p-value < 0.05 vs. wt, statistical test: Log-rank test. (**C**, **D**) Kaplan Meier curves of Abeta;wt and Abeta;ahr-1 grown on OP50. Pooled data of 120 worms/condition in 2 independent replicates are shown. Statistical evaluation with log-rank test did not display significant differences.
