# Supplementary material for: Dietary and environmental factors have opposite AhR-dependent effects on C. elegans healthspan
Source: Aging (Albany NY). 2020 Dec 13;13(1):104–33. doi: 10.18632/aging.202316 (PMC7835051; doi:10.18632/aging.202316)
Supplement: Supplementary Tables [file aging-13-202316-s003.pdf]

SUPPLEMENTARY TABLES

Supplementary Table 1. Relative expression of transgenes in control or *ahr-1* RNAi-treated reporter strains.

Strain name	Gene	Rel. expression (mean ± SD)	N	n	p-value
BC14926	**cyp-14A3**	anterior: 0.97<br>posterior: 1.05	38 (con)<br>35 (ahr-1)	3	0.544<br>0.333
SD1444	**cyp-25A2**	1.07 ± 0.16	38	2	0.067
BC20334	**cyp-29A2**	0.85 ± 0.60	42	2	0.377
cyp-35A2	**cyp-35A2**	anterior: 8.43 ± 11.96<br>posterior: 1.99 ± 2.14	43 (con)<br>42 (ahr-1)	3	< 0.001<br>0.005
cyp-35A3	**cyp-35A3**	1.08 ± 0.09	28 (con)<br>26 (ahr-1)	2	0.02
CY573	**cyp-35B1**	anterior: 2.60 ± 1.97	36 (con)<br>27 (ahr-1)	2	< 0.001
		posterior: 2.90 ± 2.40	55 (con)<br>44 (ahr-1)	3	< 0.001
BC15044	**cyp-37A1**	0.66 ± 0.14	46 (con)<br>26 (ahr-1)	2	< 0.001
CL2166	**gst-4**	1.58 ± 0.37	27 (con)<br>29 (ahr-1)	2	< 0.001
ugt-29	**ugt-29**	0.96 ± 0.05	44 (con)<br>36 (ahr-1)	3	0.004

The relative expression was measured in worms on their first day of adulthood and normalized to control RNAi treated worms in each replicate. N shows the number of worms and n the number of experiments, Statistical test: 2-tailed unpaired t-test.

Supplementary Table 2. *C. elegans* strains used in this study.

Strain name	*Genotype*
**BC14926**	*dpy-5(e907); sEx14926[rCes K09A11.4::GFP + pCeh361]*
**BC15044**	*dpy-5(e907); sEx15044[rCes F01D5.9::GFP + pCeh361]*
**BC20306**	*cyp-34A9p::GFP*
**BC20334**	*cyp-29A2p::GFP*
**CL2166**	*pAF15)gst-4p::GFP::NLS*
**CY573**	*cyp-35B1p::GFP + gcy-7p::GFP*
**cyp35A2**	*cyp-35A2p::GFP*
**cyp35A3**	*cyp-35A3p::GFP*
**cyp35A5**	*cyp-35A5p::GFP*
**cyp33E2**	*cyp-33E2p::GFP*
**CZ2485**	*ahr-1(ju145)*
**N2**	*wild-type*
**NL2098**	*rrf-1(pk1417)*
**NL2550**	*ppw-1(pk2505)*
**NR222**	*rde-1(ne219); kzIs9[lin-26p::nls::GFP + lin-26p::rde-1 + rol-6(su1006)]*
**NV33a**	*cyp-35B1p::GFP + gcy-7p::GFP; ahr-1(ju145)*
**NV33wt**	*cyp-35B1p::GFP + gcy-7p::GFP*
**NV35a ***	*ahr-1(ju145); (pAF15)gst-4p::GFP::NLS*
**NV35wt ***	*pAF15)gst-4p::GFP::NLS*
**NV38b ***	*ahr-1(ju145); unc-54p::Q40::YFP*
**NV38wt ***	*unc-54p::Q40::YFP*
**NV41a ***	*unc-119p::Aβ 1–42; Pmyo-2::YFP; ahr-1(ju145)*
**NV41wt ***	*unc-119p::Aβ 1–42; Pmyo-2::YFP*
**NV42a ***	*unc-54p::alphasynuclein::YFP, ahr-1(ju145)*
**NV42wt ***	*unc-54p::alphasynuclein::YFP*
**NV47a ***	*ugt-29p::GFP; ahr-1(ju145)*
**NV47wt ***	*ugt-29p::GFP*
**SD1444**	*unc-119(ed3); gaIs237 [cyp-25A2p::his-24::mCherry + unc-119(+)]*
**TU3311**	*uIs60[unc119p::YFP; unc119p::sid-1]*
**TU3401**	*sid-1(pk3321); uls69[myo-2p::mCherry + unc-119p::sid-1]*
**ugt-29**	*ugt-29p::GFP*
**VP303**	*rde-1(ne300); neIs9 [myo-3::HA::RDE-1+ rol-6(su1006)]*
**WM118**	*rde-1(ne219); kbIs7 [nhx-2p::rde-1 + rol-6(su1006)]*
**ZG24**	*ahr-1(ia03)*

Strains are sorted alphabetically.
* these strains where constructed using classical breeding techniques.

Supplementary Table 3. Primer pairs used in this study to validate genes differentially expressed between *ahr-1(ju145)* and wild-type (Supplementary Figure 3).

Gene		Sequence (5‘ – 3‘)	Annealing temp. (°C)	Efficiency (%)
clec-209	F	TGCTCGGGGAACAACCAAAA	60	88.4
	R	TTGGCTACGAACGATTGATGC		
C01B4.6	F	TGGCGATGCGAAAATTGATGTAA	61	83.9
	R	ATCTCCAGAAAGTGCTCGGC		
F56A4.3	F	ACGAGGGGAATGAATGGCAA	67	101.4
	R	CCATAGGGACCAATATCCATGAACT		
C01B4.7	F	GTTTTGGAATCAGACGCGGG	67	106.6
	R	CAGTGGGGTTCCGTCAAGTT		
atf-2	F	CGAAGGAACAATGAAGCCGC	67	98.1
	R	CCAAGAGCTGAACTCGTCGT		
K04H4.2	F	ACGCCGGAATCTGTTGTTCT	67	95.3
	R	CGTTCATTTGGAAAGGAGGCAT		
egl-46	F	CACCTCAACCGCTTTTCCAAG	67	85.1
	R	ATTTCACATCCGCCTCCTCC		
T20F5.4	F	TCATCTACCGAGCAGCCAAC	61	94.5
	R	GAGATGCTCGGTCTCACTGC		
ptr-4	F	TCCTACCAGACGCGCAATC	66	79.5
	R	GCAACCCATACTGACGGAGT		
dyf-7	F	GTCTGCGTTTCCGTCACAAG	66	124.8
	R	CGGGGAAGCAACAAGTTCTG		
cdc-42	F	ATTACGCCGTCACAGTAATG	62	100.8
	R	ATCCCTGAGATCGACTTGAG	58	107.2
act-1	F	GCTCTTGCCCCATCAACCAT	60	99.7
	R	CACTTGCGGTGAACGATGGA	58	98.1
